# Historical development of ADHD from 1753 to 2026

**DOI:** 10.3389/fpsyg.2026.1938091

**Published:** 2026-09-14

**Authors:** Farzad Salehpour, Francisco Gonzalez-Lima

**Affiliations:** 1 Texas Consortium in Behavioral Neuroscience, Department of Psychology, The University of Texas at Austin, Austin, TX, United States; 2 Interdisciplinary Neuroscience Program, Department of Psychiatry and Behavioral Sciences, Dell Medical School, The University of Texas at Austin, Austin, TX, United States

**Keywords:** ADHD, diagnostic evolution, executive dysfunction, history of ADHD, hyperactivity, impulsivity, inattention, neurobiology of ADHD

## Abstract

**Introduction:**

The conceptualization of attention-deficit/hyperactivity disorder (ADHD) has evolved from early moral interpretations into a complex, multidimensional neurodevelopmental syndrome. This review traces the progressive convergence of clinical observation, developmental psychopathology, genetics, and behavioral neuroscience across history.

**Methods:**

We synthesized historical literature, clinical milestones, neuroimaging data, and molecular genetics from the 18th century to the present. The evolution of diagnostic terminology and successive revisions of the Diagnostic and Statistical Manual of Mental Disorders (DSM) were evaluated alongside emerging therapeutic trends and computational models.

**Results:**

Early 20th-century observations of encephalitis lethargica shifted the field toward neurobiological paradigms. Catecholaminergic theories and family studies in the 1970s and 1980s established biological underpinnings, moving focus from mere hyperactivity to core deficits in executive functioning. The subsequent neuroscience-driven era used structural and functional neuroimaging to implicate fronto-striatal, fronto-parietal, and cerebellar circuits. Distributed functional networks have replaced single-deficit models, while large-scale genome-wide association studies suggest that ADHD may be highly polygenic. Concurrently, therapeutics have expanded beyond stimulants to non-stimulants, behavioral therapies, and non-invasive neuromodulation techniques like prefrontal photobiomodulation.

**Discussion:**

Despite significant progress, critical gaps remain. Future research must transition from categorical diagnostic models to dimensional, biologically informed frameworks that capture individual heterogeneity. Identifying reproducible biomarkers through multimodal data integration and machine learning is a high priority. Furthermore, longitudinal studies are essential to clarify lifespan trajectories, symptom persistence into adulthood, and late-life manifestations. Improving sample representativeness by actively including females, adults, and ethnically diverse populations is vital for generalizability. Finally, investigating overlapping or distinct non-hyperactive phenotypes, such as cognitive disengagement syndrome, will refine future nosology. An interdisciplinary, precision psychiatry approach is necessary to transform ADHD from a symptom-based diagnosis into a personalized, biologically informed neurodevelopmental disorder.

## 1 The 18th century: early medical descriptions of inattention and hyperactivity

On March 18, 2024, van Kernebeek and Crunelle published a research article in the *Journal of Attention Disorders*, which identified some of the earliest known references to what is now recognized as attention-deficit/hyperactivity disorder (ADHD) dating back to 1753 (van Kernebeek and Crunelle, 2024). In that year, the term “Attentio Volubilis” (Latin for “Fleeting Attention”) was used in the Dutch psychiatry treatise *Treatise on the Diseases of the Soul Caused by Weakened Movement of the Cerebral Medulla* by Cornelius Albertus Kloekhof, a Dutch physician (Kloekhof, 1753). In his original 1753 treatise, Kloekhof pointed out several archaic ideas without a scientific basis: (1) that excessive irritation of nerves on both sides at the back of the head, combined with nerve weakness, would cause fleeting attention; (2) that this interference would disrupt both the consistency and clarity of ideas, which in turn would impair memory; (3) that it was crucial that good judgment resist excessive irritability of the nerves; and (4) that if the mind persistently struggled to hold on to a few ideas, it could manage the changes in the nerves that these selected ideas induce. Kloekhof's writing on attention is brief and lacks actual clinical examples. Also in 1753, a French physician, Le Camus, described attention deficit in a manner very similar to Kloekhof (Le Camus, 1753). Which of these two physicians published first is unclear. However, the work of Le Camus did not have the same influence on the history of ADHD as Kloekhof's ideas about “Attentio Volubilis” that placed impaired attention within the field of medical study (van Kernebeek and Crunelle, 2024).

In 1775, Melchior Adam Weikard, a German physician, characterized a group of children notable for their poor attention spans and their tendency to be easily distracted (Weikard, 1775). Quoting his own observations, Weikard termed their condition as “jumpiness,” a term that aligns with today's understanding of hyperactivity. He also made reference to their restlessness and impulsivity, characteristics that are currently recognized as risk-taking behavior. Importantly, Weikard acknowledged that this disorder surfaces early in life and interferes with the child's academic performance at school (Barkley and Peters, 2012). Weikard also used the term “Attentio Volubilis” when describing these children in his work (Martinez-Badía and Martinez-Raga, 2015). The identical use of this term by both Weikard and Kloekhof is not coincidental. Kloekhof's book was translated into German, and during his era, his works were widely read. It appears that Weikard was one of those who extensively engaged with his texts (van Kernebeek and Crunelle, 2024).

In 1798, Alexander Crichton, a Scottish physician, made noteworthy observations about a group of children who demonstrated an inability to consistently focus on any one task or object. Interestingly, Crichton highlighted a phenomenon he termed “mental restlessness,” a concept that aligns with contemporary research showing increased mind wandering among ADHD patients. This connection is particularly striking given that it was first described in the 18th century. Crichton observed that these children were not only easily distracted by external stimuli but also exhibited a perceived lack of mental energy to sustain attention. This can be interpreted as an early understanding of what is now referred to as arousal problems. Crichton was the first to propose that attention disorders can have multiple etiologies with neurobiological causes, which almost always arise from an unnatural or morbid sensibility of the nerves (Crichton, 1798).

Taken together, these 18th-century accounts marked an early shift from general observations of problematic behavior toward the medical conceptualization of attention and behavioral regulation. Although predating modern diagnostic frameworks, they introduced several enduring features of ADHD, including inattention, hyperactivity and impulsivity, early-life onset, functional impairment, and possible biological origins.

## 2 The 19th century: early clinical and conceptual descriptions of ADHD-like behaviors

During the early 19th century, several clinical accounts began to describe behavioral features resembling what are now recognized as core ADHD symptoms, particularly impulsivity, inattention, and poor behavioral control. In 1809, John Haslam, an English physician, reported on what appears to have been an instance of ADHD in a 10-year-old boy characterized by uncontrollable behavior, impulsiveness, and being exceedingly difficult for his family to manage (Haslam, 1809). In 1812, the renowned American physician Benjamin Rush had examined three individuals exhibiting a complete distortion of moral judgment, which encompassed an inability to sustain attention (Rush, 1812). In 1845, Heinrich Hoffman, a German pediatrician, published a book regarding the psychological states of children from his clinical observations. He depicted both a highly impulsive child named “Fidgety Phil” and a distractible, daydreaming child he referred to as “Johnny Head-in-Air” (Hoffman, 1865).

As the century progressed, clinical descriptions increasingly broadened beyond isolated symptoms toward concepts involving attentional control, behavioral instability, and self-regulation. In 1845, Jean-Etienne Dominique Esquirol, a French psychiatrist, might have laid the groundwork for identifying ADHD through his observations of children and adults struggling with attention difficulties, and stated that those affected by insanity lose the ability to concentrate and steer their attention effectively (Esquirol, 1845). Alternatively, the origins of ADHD in French medical history might trace back to the concept of “mental instability” documented in French medical texts between 1885 and 1895, spearheaded by (Bourneville 1888, 1895) at the Hospital Bicêtre in Paris. Bourneville noted observations of children and adolescents deemed “abnormal” and placed in medical and educational facilities, many of whom displayed issues with attention and other behavioral challenges (Bourneville, 1895). In 1892, Charles Baker, a student of Bourneville, provided a clinical report of four children exhibiting hyperactivity and impulsiveness as part of his dissertation, including an emphasis on attentional difficulties in one case. In 1867, Henry Maudsley, an English psychiatrist, wrote about a child characterized by impulsivity and a propensity for destructiveness (Maudsley, 1867). In 1890, William James, an American psychologist, mentioned in his “*Principles of Psychology”* a personality type he termed “explosive will,” similar to the challenges faced by individuals now identified with ADHD (James, 1890).

By the end of the 19th century, the literature also began to extend beyond clinical description toward early approaches to management. In 1899, Thomas Clouston, a Scottish psychiatrist, published a notable paper on the behavior of impulsive children with learning problems. In his paper, he suggested a therapy comprising individualized dietary and social treatments, which included environmental enhancements, as well as a pharmacotherapy regimen that involved doses of potassium bromide, an anticonvulsant first used in 1857 for epilepsy treatment in humans (Clouston, 1899).

Collectively, 19th-century accounts broadened the understanding of ADHD-like behaviors from isolated symptoms toward more integrated clinical concepts involving attention, impulsivity, and behavioral self-regulation.

## 3 1900 to 1960

### 3.1 Early clinical and neurological conceptualizations

In 1902, through a set of three lectures presented to the Royal College of Physicians, George Frederic Still detailed his observations of 43 children from his clinical practice who struggled with sustained attention. Echoing the thoughts of American psychologist William James, he believed that such attention could play a crucial role in the “moral control of behavior.” Consequently, Still referred to the condition as “the disease of moral control.” Despite this anachronistic term, Still is recognized as the first British pediatrician, and he emphasized the emotional regulation difficulties experienced by those children. These issues had been largely overlooked until they started garnering more attention in recent times. Still's focus was on issues like immediate gratification and a severe inability to manage emotional activities, terms that correspond with what modern psychologists define as emotional dysregulation. Furthermore, Still observed that these patients had little control over their behavior and experienced problems with sustained attention. His comments about these individuals acting without considering the consequences reflect difficulties with forward-thinking or temporal foresight, processes now known to be largely mediated by the frontal lobes (Still, 1902). Drawing from the theories of William James, Still proposed that the lack of sustained attention, moral control, and inhibitory volition were interlinked and stemmed from a common neurological deficiency (Barkley, 2015). This interpretation marked an important conceptual transition from predominantly moral and behavioral explanations toward neurological hypotheses of ADHD-like symptoms. Still further speculated that this could involve “cell-modification dependent upon interference with cell-nutrition,” one of the earliest suggestions of a biochemical basis for childhood behavior disorders (Still, 2006).

Building on Still's observations, Alfred Frank Tredgold argued in 1908 that early brain damage, including that associated with birth defects or perinatal anoxia, could contribute to later behavioral or learning disabilities (Tredgold, 1922). This connection was further supported by the global encephalitis lethargica epidemic from 1917 to 1928, which impacted around 20 million individuals (Rafalovich, 2001). Indeed, the focus on ADHD in North America is believed to have also originated from this epidemic in 1917, during which clinicians encountered numerous children who exhibited significant behavioral and cognitive aftereffects. The residual effects were described as a “post-encephalitic behavior disorder” which was clearly a result of brain damage.

In 1917, the physician Rodriguez-Lafora in Spain identified a group of children with psychopathic traits, specifically naming a portion of them as the “unstable.” His characterization of these children aligns closely with today's understanding of ADHD, highlighting symptoms such as attention deficit, heightened activity, and impulsive actions. He also noted that these children tend to be overwhelmed by their adventurous nature (Lafora, 1917; Bauermeister and Barkley, 2010).

In 1923, Franklin Ebaugh was among the first clinicians to comprehensively characterize the behavioral sequelae of post-encephalitis. His analysis was based on 17 children referred to his clinic at Philadelphia General Hospital who had survived the encephalitis epidemic (Ebaugh, 1923). Commonly noted characteristics encompassed drastic personality shifts, emotional volatility, cognitive impairments, learning challenges, sleep disturbances, tics, depression, and subpar motor skills. These children frequently displayed hyperactivity, distractibility, irritability, antisocial behaviors, destructiveness, unruliness, and had difficulties at school. They often disrupted their peers and were seen as argumentative and impulsive, sometimes even exiting the school premises during lessons without consent. These descriptions of cognitive and behavioral abnormalities were also highly similar to those reported earlier by Still (1902). Ebaugh observed that 10 of these children exhibited acute post-encephalitic behavioral changes despite being behaviorally normal before the pandemic, while the remaining seven appeared to have preexisting behavioral issues that were worsened by encephalitis. These findings led Ebaugh to suggest, for the first time, that while ADHD-like symptoms could arise acutely following brain injury (e.g., encephalitis), in some cases, such symptoms may also represent preexisting problems exacerbated by the injury (Barkley, 2007).

In 1925, Sue Stryker also described the behavioral sequelae of post-encephalitis, many of which closely resembled symptoms now associated with ADHD (Stryker, 1925). Although these post-encephalitic cases could be linked to ADHD based on their clinical presentation, the majority would not meet modern diagnostic criteria (Lange et al., 2010). Symptoms that are currently labeled as oppositional defiant disorder (ODD), along with delinquency and conduct disorder (CD), were also observed in certain instances (Barkley, 2015). The behavioral symptoms observed in post-encephalitic children in the 1920s further confirmed the concept of organicity (bodily basis), indicating that brain-based damage can be associated with ADHD-like behaviors (Baumeister et al., 2012).

Further neurological interpretations emerged in 1926, when Bond and Partridge suggested a link between post-encephalitic hyperkinetic behavioral syndromes and functional disturbances in the basal ganglia, particularly the corpus striatum (Bond and Partridge, 1926).

By 1932, Franz Kramer and Hans Pollnow had provided an extensive clinical characterization of what is now recognized as the “hyperkinetic disorder” (Kramer and Pollnow, 1932). Their descriptions highlighted features such as hyperkinesis, inattention, and impulsiveness, aligning with our current understanding of the condition (Sadek and Sadek, 2014). During the 1930s, there was a significant realization about the marked resemblance between children's hyperactivity and the behavioral outcomes observed in primates with frontal lobe damage (Levin, 1938). Earlier studies from the 1870s had revealed that removing the frontal lobe in monkeys led to symptoms like extreme restlessness, diminished concentration, aimless movement, and increased appetite, among other behavioral shifts (Ferrier, 1876). Therefore, multiple researchers, including Levin (1938), pointed out these similarities to suggest that extreme restlessness in children could potentially stem from abnormalities in the forebrain structures. However, clear signs of such defects were not consistently visible in many of these children. During this period, milder cases of hyperactivity were often linked to psychological reasons, like indulgent parenting or negative family surroundings.

In 1934, Kahn and Cohen significantly contributed to the development of the concept of ADHD as a neurobiological disorder by proposing the existence of a syndrome called “organic drivenness,” characterized by a surplus of inner impulsion, general hyperkinesis, difficulty maintaining quiet behavior, and extreme fluctuations in attention (Kahn and Cohen, 1934). They also suggested, based on von Economo's findings (Von Economo, 1931), that damage to the brainstem (e.g., due to encephalitis) could lead to organic drivenness. This indicates that, from Kahn and Cohen's perspective, organic drivenness was not solely the result of encephalitis but could also arise from other potential biological etiologies (Baumeister et al., 2012).

### 3.2 Emergence of stimulant treatment and child psychopharmacology

From 1937 to 1941, a pivotal series of articles emerged, marking the onset of medication therapy, especially stimulants, for behaviorally disordered children and laying the foundation for the field of child psychopharmacology (Baumeister et al., 2012).

In 1937, a significant moment in ADHD history took place when the physician Charles Bradley, based at Rhode Island, United States, conducted studies where he extracted cerebral spinal fluid from adolescents displaying severe behavioral issues. Many of these descriptions suggest that these youngsters might have had ADHD. To address the side effects of these spinal procedures, Bradley administered the stimulant Benzedrine to the children, originally to alleviate their headaches. Surprisingly, he observed a marked improvement in their behavior (Bradley, 1937; Baumeister et al., 2012).

In 1937, another major contribution to early ADHD-related diagnostic research emerged from Bradley's work (Bradley, 1937). As part of the clinical evaluation, Bradley employed the newly developed electroencephalographic (EEG) technology (Berger, 1929) in 20 of the 30 children who participated in the Benzedrine trial. Among those 20, EEG recordings revealed that 11 children exhibited definite abnormalities in brain rhythms. Of these 11, eight showed a “spike and slow wave” pattern, a signature commonly associated with petit mal epilepsy. Benzedrine appeared to subdue behavior in seven of these eight children. Bradley interpreted these EEG abnormalities as indicative of impaired cortical function and proposed that the observed behavioral improvements may have resulted from stimulant-induced activation of inhibitory cortical systems (Bradley, 1937).

An important pharmacological development occurred in 1944, when Leandro Panizzon, an Italian chemist, synthesized methylphenidate in Basel while working for the Swiss pharmaceutical company CIBA (now Novartis) (Panizzon, 1944). Bradley named the drug after his wife Margarita, nicknamed Rita. While Leandro was not overly impressed by the results of a self-experiment, his wife enjoyed the stimulating effects of the substance before playing tennis (Zimmer, 2017). By 1954, CIBA had launched Ritalin in Germany and Switzerland, advertising it as a psychotonicum for alleviating chronic fatigue and mild depression (Lange et al., 2010). In 1955, Ritalin was also marketed for narcolepsy. In 1956, physicians started prescribing Ritalin for children's behavior problems (Singh, 2007) based on earlier evidence provided by Charles Bradley, an American psychiatrist, on the use of psychostimulant drugs for ADHD treatment, such as Benzedrine (Wood et al., 2014). Ritalin was not reported to be a stimulant until 1957 (Wood et al., 2014). Subsequent research guided by Bradley's original findings affirmed that over half of the hospitalized hyperactive children responded positively to stimulants.

### 3.3 From brain injury to hyperkinetic syndromes

In the 1940s, a common perspective emerged suggesting that many children admitted to psychiatric hospitals displaying these symptoms might have experienced some form of brain injury (like encephalitis or prenatal-perinatal trauma), even if their clinical history did not necessarily show evidence of this.

In 1947, the notion of the “brain-injured child” emerged and was attributed to children exhibiting these behavioral traits, even though many did not have proof of brain abnormalities (Strauss and Lehtinen, 1947). During the 1940s, partly due to the lack of concrete evidence of brain damage, the terminology shifted from “brain-injured child” to “minimal brain damage” and remained in use until the late 1950s (Barkley, 2015).

In 1952, the first edition of the Diagnostic and Statistical Manual of Mental Disorders (DSM-I) (American Psychiatric Association, 1952) did not include a distinct diagnostic category for the constellation of symptoms now associated with ADHD, nor did it provide a formalized coding index for such symptoms.

In 1957, Laufer et al. conducted a photo-Metrazol EEG study in children with hyperkinetic syndrome and found significantly lower thresholds on the photo-Metrazol test compared to healthy controls, whereas amphetamine raised the threshold in hyperkinetic children (Laufer and Denhoff, 1957). Based on findings recently reported by Gastaut (1950), they proposed that this low photo-Metrazol threshold indicates dysfunction in subcortical structures in early life, particularly the diencephalon. Furthermore, drawing on the work of Bradley and Elkes (1953) and Bradley and Elkes (1957) who showed that the site of action of amphetamine may involve the brain's reticular activating system, and Magoun (1952), who described this system as an ascending arousal network extending from the brainstem to the diencephalon and then to the cortex, they hypothesized that in the hyperkinetic syndrome of behavior, dysfunction of the diencephalon may expose the cortex to abnormally intense sensory input from peripheral receptors. This excessive input, transmitted via the diencephalon and the reticular activating system, may disrupt cortical function and impair cortex-diencephalon interactions. In this paper, the term “Hyperkinetic Impulse Disorder” was introduced for the first time, coined by Laufer et al. to describe the behavioral syndrome they were investigating (Laufer and Denhoff, 1957).

In 1957, Laufer and Denhoff also published the first report on the potential association between sleep disturbances and hyperkinetic behavior, stating that

“*Generally, the parents of hyperkinetic children are so desperate over the night problems that the daytime ones pale in significance*” (Laufer and Denhoff, 1957).

Overall, the first half of the 20th century marked a major shift from moral and descriptive interpretations toward biological and neurological explanations of ADHD-like behaviors. Evidence from post-encephalitic syndromes, emerging brain-based models, and the therapeutic effects of stimulants increasingly supported an organic basis for the disorder, while the transition from brain injury to minimal brain damage and hyperkinetic concepts laid the groundwork for later symptom-based classifications.

## 4 The 1960s

### 4.1 Decline of minimal brain dysfunction and emergence of hyperactivity

By the 1950s−1960s, criticism mounted that the term “Damage” in “Minimal Brain Damage” was too strong, as some cases did not show clear neurological deficits responsible for the observed behavioral abnormalities. As an alternative, the U.S. Public Health Service and affiliated experts suggested the term “Minimal Brain Dysfunction” to reflect functional deficits without confirmed structural lesions (Clements, 1966). This change represented a broader conceptual shift from presumed structural brain injury toward functional neurological dysfunction in the absence of demonstrable lesions. However, even this revised concept soon came under scrutiny. During the 1960s, many critiques began challenging the validity of minimal brain dysfunction as a singular syndrome in children, leading to a surge in research on this population (Rie, 1980). Over time, the concept of minimal brain dysfunction gradually declined due to its ambiguity, broad scope, lack of diagnostic clarity, and weak neurological foundation. Eventually, terms like “dyslexia,” “language disorders,” “learning disabilities,” and “hyperactivity” took its place. These new terms focused on clear and observable children's deficits rather than an unseen cause within the brain.

In 1960, Stella Chess coined the term “Hyperactive Child Syndrome” and defined hyperactivity as:

“*The hyperactive child is one who carries out activities at a higher-than-normal rate of speed compared to the average child, or who is constantly in motion, or both*.”

This definition was historically groundbreaking in the understanding of hyperactivity (Chess, 1960). Chess shifted focus to the observable rate of activity and consistent motion as the main characteristics, moving away from other contemporaneous scientists' conjectures about potential neurological causes. Apparently, this perspective was informed by her clinical experience; for example, in Chess's well-known report on 82 hyperactive children, only 14 were diagnosed as brain-damaged (Chess, 1960). Furthermore, she emphasized the significance of objective evidence in diagnosing the symptom, advocating for a reliance on more than just the anecdotal reports of parents or teachers. Her perspective also alleviated parents from bearing the blame for their children hyperactive behavior. Additionally, Chess distinctly separated the idea of hyperactivity from the broader and more nebulous concept of a brain damage syndrome, refining the understanding and diagnosis of hyperactivity in children (Treloar, 1992).

In 1963, Kirk and Becker (1963), followed by Birch (1964) and Herbert (1964) in 1964, further criticized the concept of minimal brain dysfunction as over-inclusive, of little prescriptive value, and lacking considerable neurological evidence.

Concern regarding the terminology and identification of minimal brain dysfunction led the National Institute of Neurological Diseases and Blindness to convene a conference in 1963, which subsequently resulted in the formation of a national task force (Kessler, 1980; Lange et al., 2010). In 1966, the task force recognized at least 99 symptoms associated with the disorder and published its official definition and recommendations as follows:

“*The term minimal brain dysfunction refers to children of near average, average, or above average general intelligence with certain learning or behavioral disabilities ranging from mild to severe, which are associated with deviations of function of the central nervous system. These deviations may manifest themselves by various combinations of impairment in perception, conceptualization, language, memory and control of attention, impulse or motor function”* (Clements, 1966).

This definition has two important aspects. First, it has been suggested that the concept of the three main symptoms of inattention, impulsivity, and hyperactivity, which are now central to ADHD, was established in this definition, as the task force explicitly grouped these three together at the end of the statement. Secondly, by this definition, the task force assigned individuals with minimal brain dysfunction to the normal range of general intelligence, distinguishing them from those described as brain-damaged or mentally subnormal (Lange et al., 2010).

During the 1960s Chess and other scientists also promoted an emphasis on the symptoms of hyperactivity as relatively mild or “benign.” These researchers posited that, in most cases, hyperactivity symptoms would fade and resolve by puberty. Stemming from these claims, a dominant belief took shape and continued into the 1980s, that hyperactivity (later termed ADHD) was a condition children would naturally outgrow by adolescence.

During the 1960s or even earlier, the viewpoint on hyperactivity in North America was different from that in Europe, particularly in Great Britain. In North America, hyperactivity was seen as heightened activity, a common childhood issue in need of medication, not always linked to brain pathology or intellectual disability. In Great Britain through the 1970s, hyperactivity or hyperkinesis was perceived as an extreme state of excessive activity. This uncommon condition was often associated with signs of brain damage, such as epilepsy and was frequently linked to previous brain injuries from trauma or infection. The differing perspectives resulted in significant disparities between North American and European estimates of the prevalence of the disorder, the diagnostic standards, and favored treatment approaches. It was not until deep into the 1980s that these views began to converge (Rutter, 1989).

A major diagnostic transition followed in 1968, when the DSM-II introduced hyperactivity into official diagnostic nomenclature, referring to the disorder as “Hyperkinetic Reaction of Childhood (or Adolescence)” under the category of “Behavior Disorders of Childhood and Adolescence” to emphasize hyperactivity as the primary behavioral deficit. DSM-II described the disorder as follows:

“*The disorder is characterized by overactivity, restlessness, distractibility, and short attention span, especially in young children; the behavior usually diminishes by adolescence”* (American Psychiatric Association, 1968), reflecting Chess's (1960) perspective.

By the late 1960s, amid growing discontent with the term minimal brain dysfunction and increasing uncertainty about its association with brain damage, clinical psychologists began to focus more explicitly on hyperactivity, which was increasingly regarded as the defining symptom of the disorder now known as ADHD.

### 4.2 Emerging genetic evidence and early pharmacotherapy research in hyperactivity

In 1961, Ritalin (or generic methylphenidate) became the first stimulant to be approved by the U.S. Food and Drug Administration (FDA) for use in children with behavioral problems (Mayes and Rafalovich, 2007). In 1963, Conners and Eisenberg published the first report on the therapeutic benefits of Ritalin in 81 hyperactive children. Their study was pioneering in ADHD pharmacotherapy research, as it employed a double-blind, sham-controlled design, and uniquely relied on symptom ratings from parents and childcare workers rather than clinicians (Conners and Eisenberg, 1963).

Genetic evidence also began to emerge during this period. In 1965, Rafael Lopez published the earliest twin study on the genetic and congenital contributions to hyperactivity, which examined concordance rates in monozygotic twins compared to dizygotic twins and found notably higher concordance in monozygotic twins (Lopez, 1965).

In 1967, the National Institute of Mental Health (NIMH) funded the first federal research grant to study the efficacy of stimulants in children with behavioral problems, awarding it to Keith Conners at Massachusetts General Hospital. The year 1968 is considered a turning point in pediatric psychopharmacology research, as three additional grants were awarded to Lawrence Greenberg at the University of California, Davis; Grace Steinberg at the Department of Mental Health in the District of Columbia; and Donald Klein at Hillside Hospital in New York, to investigate the “pharmacotherapy of hyperactive children” and the “comparative drug effects in hyperkinetic children” (Swanson et al., 1995). By the end of the 1960s, approximately 200,000 children in the U.S. were being treated with stimulant medications (Mayes and Rafalovich, 2007).

In sum, the 1960s represented a transition from poorly defined concepts of brain damage and dysfunction toward more observable and operationalized features of hyperactivity. The introduction of hyperactivity into formal diagnostic nomenclature, together with emerging genetic evidence and more systematic pharmacotherapy research, helped establish ADHD-like behavior as a more clearly defined clinical and research construct.

## 5 The 1970s

### 5.1 Emerging genetic and neurotransmitter foundations of hyperactivity

In 1970, Conan Kornetsky proposed the first neurotransmitter-based theory of hyperactivity. He suggested that the action of amphetamine involves the inhibition of norepinephrine synthesis, turnover, or release, thereby establishing the first catecholamine theory of hyperactivity. Based on his research on amphetamine use in children, Kornetsky proposed that hyperactivity might stem from dysregulation in catecholaminergic neurotransmission, particularly involving norepinephrine (Kornetsky, 1970).

During the 1970s, converging family and twin studies provided some of the earliest empirical evidence that hyperactivity clustered within families and might have a heritable or congenital component. Morrison and Stewart reported similar symptoms in 20% of parents of hyperactive children, as well as elevated rates of antisocial behavior, alcohol abuse, and hysteria among parents (Morrison and Stewart, 1971), findings subsequently supported by Cantwell (1972). Welner et al. (1977) likewise reported hyperactivity in 17% of siblings of affected children. Complementing these family studies, Willerman (1973) found substantially higher correlations in activity levels among monozygotic than dizygotic twins, further supporting a significant heritable or congenital contribution to hyperactivit.

In 1975, Roberts et al. conducted a pivotal animal study using a rat lesion model to investigate the neurochemical mechanisms underlying the effects of stimulant medications. Specifically, they examined amphetamine-induced hyperactivity, which is widely used as a proxy for dopaminergic activation, in order to determine how different neurotransmitter systems contribute to the locomotor-stimulating effects of amphetamines. By selectively disrupting ascending dopaminergic and noradrenergic pathways, they demonstrated that hyperactivity was critically dependent on dopamine transmission but not norepinephrine (Roberts et al., 1975). This study provided some of the earliest experimental evidence linking dopamine dysregulation to the pathophysiology of hyperactivity. It also reinforced Kornetsky's earlier catecholamine hypothesis, which framed hyperactivity as a disorder of catecholaminergic imbalance, particularly involving norepinephrine (Kornetsky, 1970).

In 1977, Alpern and Greer employed a genetically selected mouse model to simulate core behavioral features of preadolescent hyperactivity. Their findings further supported the dopaminergic basis of stimulant effects, showing that the therapeutic (anti-excitatory) actions of amphetamine in hyperkinetic behavior are likely mediated by dopamine. As they concluded,

“*Decidedly, the most important finding to emerge in this study is the clear indication that amphetamine's attenuation of neural excitability, indexed by myoclonic onset, might be mediated by dopamine*.” (Alpern and Greer, 1977).

### 5.2 Emerging neuropsychological models of hyperactivity and attention

By the early 1970s, the characteristics considered central to hyperactivity or hyperkinesis expanded to encompass traits that researchers had once thought were merely related, such as impulsivity, limited attention span, reduced frustration tolerance, distractibility, and aggression. These developments began to shift the conceptual emphasis beyond hyperactivity alone toward deficits in sustained attention and impulse control as potentially more central features of the disorder. The 1970s were marked by two contrasting ADHD models: Paul Wender's “Model of Minimal Brain Dysfunction” and Virginia Douglas's “Model of Attention and Impulse Control.”

Within this evolving conceptual framework, Wender proposed a model of minimal brain dysfunction in 1971 to explain the core symptoms observed in affected children. He proposed that six areas of dysfunction (hyperactivity and poor motor coordination, short attention span and poor concentration, poor impulse control, learning difficulties, problems with interpersonal relations, and emotional difficulties) could be explained by three main factors: (1) a reduced experience of pleasure and pain, (2) a consistently elevated yet poorly regulated level of activation, and (3) extraversion (Wender, 1971). Wender argued that reduced sensitivity to pleasure and pain could diminish responsiveness to reward, punishment, and social influence, whereas poorly regulated activation could contribute to hyperactivity, poor inhibition, sustained attention difficulties, distractibility, and emotional hyper-reactivity. Limited frustration tolerance, anger, and temper tantrums were likewise attributed to inadequate regulation of activation. While Wender appeared to combine features of ODD (and possibly CD) with those of ADHD within a single disorder, later evidence established ADHD and ODD as distinct conditions (Hinshaw, 1987).

In 1971, Campbell, Douglas, and Morgenstern demonstrated notable problems in impulse control and cognitive styles characterized by field dependence among hyperactive children, suggesting that these children may be heavily influenced by their surroundings and could struggle to focus or distinguish specific details from the broader context (Campbell et al., 1971).

In 1972, Douglas and her research team consistently showcased that children with hyperactivity encountered their most significant challenges on tasks evaluating vigilance or sustained attention, such as the CPT. Douglas also remarked on the potential link between attention-impulse control deficits and shortcomings in moral development among her participants, particularly as they reached adolescence. Douglas posited that impaired sustained attention and impulse control were more probable causes for the challenges observed in ADHD children than mere hyperactivity, a conceptualization later referred to as the model of attention and impulse control (Douglas, 1972).

In 1973, Douglas and Campbell also showed that hyperactive children weren't necessarily more prone to distractions than their non-hyperactive counterparts. Moreover, they found that issues with prolonged attention could arise even in settings devoid of notable distractions (Campbell, 1973).

### 5.3 Etiological debates and competing explanations of hyperactivity

During the 1970s, competing explanations of hyperactivity included neurological and neuroanatomical dysfunction on the one hand, and educational, psychosocial, and environmental factors on the other.

In 1973, Anneliese Pontius proposed that many cases with minimal brain dysfunction, exhibiting hyperactive and impulsive behaviors, could be attributed to dysfunctions in the frontal lobe and caudate. This could lead to difficulties in forward planning and modifying ongoing activities (Pontius, 1973).

In 1978, Hastings and Barkley reported psychophysiological evidence suggesting brain underactivity, particularly in functions associated with the frontal lobes, in individuals with hyperactivity (Hastings and Barkley, 1978).

In 1978, Bergström and Bille published the first study to apply computed tomography (CT) in investigating brain structure in 46 children diagnosed with minimal brain damage. Fifteen children (32.6%) showed abnormal CT findings, including cerebral atrophy, asymmetries, dilated ventricles (particularly unilateral or frontal horn dilation), widened sulci or fissures, and anomalies such as an arachnoid cyst or absence of the septum pellucidum. Most abnormalities were observed in boys, with some exhibiting multiple concurrent structural changes. These findings supported the utility of CT in detecting subtle neuroanatomical irregularities in minimal brain damage, marking an early step in ADHD-related neuroimaging research (Bergström and Bille, 1978).

In 1975, Conrad argued that in most cases of hyperactivity, labeling the condition as a neurological disease was largely a myth created by intolerant teachers and parents, together with an inadequate educational system (Conrad, 1975).

Around the mid-1970s, both psychoanalysts (Bettelheim, 1973) and behaviorists (Willis and Lovaas, 1977) believed poor parenting contributed to hyperactivity. Psychoanalysts argued parents' intolerance led to heightened hyperactivity, while behaviorists pointed to ineffective conditioning resulting in non-compliance and hyperactivity. Both psychoanalysts and behaviorists emphasized mothers' pivotal role in causing hyperactivity, citing studies that linked negative mother-child interactions during preschool years with persistent hyperactivity into late childhood and adolescence. Subsequent research aimed to demonstrate that correlational data doesn't establish that poor childrearing or negative interactions directly cause hyperactivity; they merely show an association. Researchers indicated that parents' controlling and negative behavior toward hyperactive children was more a reaction to the children's lack of self-control and inattention than its cause (Humphries et al., 1978). These studies underscored that mitigating hyperactivity with stimulants diminishes mothers' negative reactions (Barkley and Cunningham, 1979). Furthermore, the persistence of hyperactivity is connected to its intensity, independent of maternal behavior (Barkley et al., 1990).

In the mid-1970s, arguments emerged suggesting that hyperactivity results from environmental factors. For example, a prevalent view held that allergic reactions to food additives, including dyes, preservatives, and salicylates, led to hyperactivity. In 1973, Benjamin Feingold proposed that diet was responsible for over half of hyperactivity cases and that avoiding certain additives could effectively treat the condition (Feingold, 1973, 1975). In 1975, the National Advisory Committee on Hyperkinesis and Food Additives (NACHFA) was formed to assess the evidence on hyperkinesis and food additives. Their initial report found Feingold's claims mostly anecdotal and called for controlled trials to verify any scientific basis for them. Between 1975 and 1980, numerous pre-clinical and clinical research studies took place. In 1980, the NACHFA concluded that there wasn't sufficient evidence to advocate for special labeling of food additives, as Feingold had recommended, suggesting these substances had minimal impact on children's behavior (Nutrition Foundation, 1980).

By the end of the 1970s, research on hyperactivity had progressed markedly, culminating in more than 3,000 published studies. Multiple clinical and academic books were produced, especially a detailed literature analysis by Ross and Ross (1976). This subject also took center stage in select journal editions and scientific events, highlighting its evolution into an area of professional, academic, and mainstream concern.

### 5.4 Evolution of management approaches in ADHD

During the 1970s, hyperactivity was understood as a behavioral condition attributed primarily to biological rather than environmental factors (Werry et al., 1970). Therefore, stimulants gained increasing traction during this period as the preferred treatment for behavioral manifestations associated with ADHD (Barkley, 2015).

In 1975, amid growing criticism of strictly neurological interpretations of hyperactivity and increasing interest in broader educational and behavioral approaches, U.S. Public Law 94–142 mandated special education for children with various disabilities and broadened existing educational provisions. This shift increased research into behavioral modification techniques for managing disruptive classroom behaviors, particularly as alternatives to stimulant medication. Although these techniques showed promise, stimulants remained more effective (Gittelman-Klein et al., 1976). However, a growing consensus emerged that stimulants should be combined with behavioral interventions and parent training as part of a more comprehensive treatment approach.

In the late 1970s, children diagnosed with hyperactivity or attention deficits in North America would probably be diagnosed with CD in Europe and undergo psychotherapy, family counseling, and parenting skills training. Medication was generally frowned upon and rarely used in Europe. However, the perspective that attention deficits were just as crucial as hyperactivity in the disorder started to gain traction in European classifications, such as the ICD-9 (World Health Organization, 1978).

Viewed as a whole, the 1970s broadened the conceptualization of hyperactivity beyond excessive motor activity toward a more multidimensional understanding centered on sustained attention and impulse-control deficits. At the same time, emerging catecholaminergic, familial, and environmental research expanded etiological thinking, while behavioral approaches broadened management beyond medication alone; together, these developments laid important foundations for the modern conceptualization of ADHD.

## 6 The 1980s

### 6.1 Diagnostic evolution and differentiation of ADHD

During the 1980s, research on hyperactivity prioritized refining diagnostic criteria, distinguishing hyperactivity from other mental disorders, and subsequently questioning the notion that the primary feature of ADHD was an inability to sustain attention.

From 1979 to 1983, Douglas expanded and provided further evidence for her hyperactivity model, concluding that ADHD symptoms could be attributed to four primary deficiencies: (1) the management and maintenance of attention and effort; (2) the inhibition of impulsive responses; (3) the modulation of arousal levels to meet situational requirements; and (4) a marked tendency to seek immediate reinforcement (Douglas, 1980; Douglas and Parry, 1983; Parry and Douglas, 1983). Although Douglas's model was not technically a theory but rather primarily descriptive and inductively derived from a review of the existing ADHD research (Barkley, 1997), it established a new scientific benchmark in the field of hyperactivity and was perhaps the first model of ADHD. Douglas's efforts were likely a significant factor in the renaming of the condition to “Attention Deficit Disorder (ADD)” in 1980 with the publication of DSM-III (American Psychiatric Association, 1980; Anastopoulos and Shelton, 2001). This edition was the first to introduce the term “ADD” and defined two subtypes, one with hyperactivity (ADD+H) and one without (ADD–H). This marked a shift toward acknowledging attention issues as central to the disorder. The new diagnostic criteria stood out for their special focus on inattention and impulsivity as key characteristics of the disorder. Moreover, they provided more detailed symptom lists, a clear numerical threshold for symptoms, the precise age of onset guidelines, and a specified duration for symptoms. Additionally, they necessitated ruling out other childhood psychiatric conditions as better justifications for the observed symptoms (American Psychiatric Association, 1980).

In 1981, researchers aimed to differentiate between “pervasive” and “situational” hyperactivity. Pervasive was characterized by hyperactivity observed both at home and school, while situational described hyperactivity present in just one of these environments (Schachar et al., 1981).

In 1983, Michael Rutter pointed out that the real measure of ADHD's validity as a syndrome is its distinguishability from other child psychiatric disorders, including affective/mood disorders, anxiety disorders, learning disorders, and especially CD (Rutter, 1983). Consequently, during the 1980s, there was a growing emphasis on more sophisticated research designs aimed at identifying the distinct characteristics of ADHD compared to other psychiatric disorders, rather than merely contrasting it with the absence of any disorder. For instance, certain studies that made these comparisons revealed that situational hyperactivity wasn't always effective in distinguishing between psychiatric groups. However, attention problems and pervasive hyperactivity (present in both home and school settings) proved to be more reliable indicators (Barkley, 2015). Other notable comparison studies revealed that children with solely ADHD often had relatives with ADHD and faced academic challenges and dysthymia. In contrast, children with CD more commonly had relatives with CD and exhibited antisocial behavior, substance abuse, depression, and family discord. These observations prompted theories suggesting ADHD stems from a biological or neuropsychological delay, while CD results from erratic and troubled upbringing often linked to parental mental health issues (Hinshaw, 1987).

In 1983, Michael Gordon introduced a compact, computerized tool capable of administering two ADHD-sensitive tests: a Continuous Performance Task (CPT) for vigilance and impulsivity, and a Direct Reinforcement of Low Rates (DRL) test for impulse control (Gordon and McClure, 1983). This was the first objective ADHD behavioral assessment tool for children. While early studies found the DRL test promising, its inability to detect stimulant medication effects led to its reduced importance in ADHD diagnosis. Conversely, the CPT effectively differentiated ADHD children from non-ADHD groups and responded to medication. Despite warnings for further research and concerns about its high false-negative rate in diagnosing ADHD, the device gained widespread clinical use by the 1990s (Barkley, 2015).

A major diagnostic revision followed in 1987 with the publication of DSM-III-R (American Psychiatric Association, 1987), leading to a change in the nomenclature of the disorder to ADHD. The DSM-III revision included several substantial changes. Firstly, it consolidated the three symptom lists (inattention, impulsivity, hyperactivity) and their respective cutoff scores from DSM-III into a single list with one cutoff score, so that an individual met criteria if they had at least eight of the 14 possible symptoms of inattention, hyperactivity, and impulsivity. Secondly, the criteria were now grounded in empirical research from behavior rating scales of child behavior and underwent extensive field testing to assess their diagnostic accuracy. Thirdly, the revision emphasized that symptoms should be inappropriate for a child's developmental level. Fourthly, it was decided that concurrent mood disorders would not exclude an ADHD diagnosis. Additionally, DSM-III-R controversially eliminated the distinction between two types of ADD (ADD–H and ADD+H). The subtype of ADD–H was removed as a formal subtype and relegated to a vaguely defined category with no explicit diagnostic criteria, called “undifferentiated ADD,” which was listed in a separate section of the manual. As a result, the DSM-III-R was perceived to have excluded the inattentive subtype. The DSM-III-R placed ADHD within a broader category of “Disruptive Behavior Disorders” alongside ODD and CD (Spitzer et al., 1990). In the late 1980s, research emerged showing that children with ADD–H tended to be more inclined toward daydreaming, showed lower activity levels, were lethargic, and struggled academically. However, they were noticeably less aggressive and faced lesser peer rejection compared to those with ADD+H. Unfortunately, these findings came too late to be considered in the DSM-III-R (American Psychiatric Association, 1987). Therefore, the terminology used to describe what was known as ADHD in 1987 developed significantly over time.

In 1988, the development of research diagnostic criteria for ADHD culminated in an international symposium (Sergeant, 1988). A broad agreement was reached, stipulating that research participants for ADHD should meet certain criteria: (1) adults reporting activity and attention issues in at least two of three distinct settings (home, school, clinic); (2) affirmation of at least three out of four problems with both activity and attention; (3) symptom onset before the age of 7; (4) a duration of symptoms for at least 2 years; (5) notably high parent-teacher ratings in ADHD symptoms; and (6) the exclusion of autism and psychosis from diagnoses.

### 6.2 Early neuroimaging research: CT and SPECT studies

Throughout the 1980s, a series of neuroimaging studies using CT and single photon emission computed tomography (SPECT) progressively advanced the investigation of structural and functional brain abnormalities in individuals with ADHD.

In 1983, Shaywitz et al. conducted one of the earliest controlled studies using CT to examine brain structure in children diagnosed with ADD. However, their findings revealed no significant differences between the ADD and control groups, leading them to conclude that CT could not detect structural abnormalities in ADD using the techniques available at the time (Shaywitz et al., 1983).

In 1984, Lou et al. conducted the first neuroimaging study using 133Xenon inhalation SPECT to examine three-dimensional regional cerebral blood flow (rCBF) in children with ADD. They found that rCBF distribution was abnormal in both hemispheres, with all patients exhibiting hypoperfusion in the frontal white matter and seven also showing reduced perfusion in the caudate nuclei. Methylphenidate hydrochloride administration was associated with increased perfusion in central brain regions, including the mesencephalon and basal ganglia, and decreased perfusion in motor and primary sensory cortices (Lou et al., 1984). However, the findings should be interpreted cautiously because six of the 11 patients had been receiving daily methylphenidate before the study, making it difficult to distinguish disease-related differences from medication, withdrawal, or re-treatment effects (Leo and Cohen, 2009). Additional concerns included older control participants and limited quantification methods (Zametkin, 1989).

In 1986, Nasrallah et al. conducted a second controlled CT study in 24 participants diagnosed with hyperkinetic syndrome or minimal brain dysfunction. They reported cortical and cerebellar atrophy (Nasrallah et al., 1986). However, these findings were interpreted with caution, as all participants had received psychostimulant treatment, raising the possibility that the observed atrophy could represent a long-term adverse effect of medication (Leo and Cohen, 2009). Moreover, 7 of the 24 participants had a history of alcohol abuse, further complicating interpretation (Zametkin, 1989).

In 1986, Zametkin and Rapoport proposed that deficits in dopamine, norepinephrine, or both could contribute to the patterns of reduced brain activity observed in individuals with ADHD. Building on the cerebral blood flow findings reported by Lou et al. (1984), they further hypothesized that lesions in the frontal regions, particularly the prefrontal cortex (PFC), along with its connections to the mesencephalon, could account for core ADHD symptoms. They suggested that impairments in impulse control were related to frontal lobe dysfunction, attention problems were associated with brainstem abnormalities, and learning difficulties reflected disruptions in higher cortical functions (Zametkin and Rapoport, 1986).

In 1989, Lou et al. also conducted the first controlled study using ^133^Xenon inhalation SPECT that identified striatal hypoperfusion as a cardinal feature in the pathophysiology of ADHD (Lou et al., 1989).

### 6.3 Emerging etiological and pathophysiological models of ADHD

In 1982, Myers et al. hypothesized that spontaneously hypertensive rats (SHRs), a genetic model of hypertension, exhibit hyperactivity analogous to ADHD symptoms. They compared SHRs with normotensive Wistar-Kyoto (WKY) rats using open-field testing and spontaneous locomotor activity assessments. SHRs showed significantly higher activity levels than WKY rats, particularly in novel environments, supporting the model's face validity for hyperactivity. The results indicated that hyperactivity was not due to hypertension *per se* but inherent to the SHR strain. The study concluded that SHRs are a valid animal model for studying hyperactive behavior relevant to ADHD, especially for pharmacological and neurobiological investigations (Myers et al., 1982).

In 1985, Barkley and his colleagues conducted research that was the first to challenge the prevailing belief that ADHD resulted from poor parenting. They discovered that the controlling behavior exhibited by parents was largely a response to the actions of their children. When the children were administered either Ritalin or a placebo without the knowledge of their parents, the effects of the medication became apparent, influencing not only the ADHD symptoms but also the children's interactions with their parents. This led to significant improvements in parental behavior. Contrary to earlier psychoanalytic or social learning theories that placed blame on parents, it became evident that the child's behavior was influencing that of the parent. This suggests that a mother's directive approach toward children with ADHD is more a reaction to the child's behavior than its root cause (Barkley et al., 1985).

By the late 1980s, theoretical accounts were beginning to extend beyond primary attentional deficits toward motivational and reinforcement-based explanations of ADHD. In 1987, Haenlein and Caul proposed the “Motivational Model of ADHD,” suggesting that core symptoms stem from a diminished sensitivity to reinforcement rather than primary attentional deficits. Around the same time, more sophisticated studies on ADHD in children revealed that attention problems were not always evident under certain experimental conditions. These findings prompted a shift toward viewing ADHD as a disorder of motivation, in which reduced responsiveness to behavioral consequences such as reinforcement or punishment plays a central role (Haenlein and Caul, 1987). The motivational model gained support due to its ability to better explain the situational variability in attention in ADHD cases, its alignment with neuroanatomical findings showing reduced activation in brain reward systems (e.g., cortical-limbic regulating circuits), its consistency with the understanding of dopamine pathways in motor function and incentive learning, and its potential to guide treatment approaches for ADHD symptoms (Beninger, 1983).

In 1989, another twin study, which evaluated hyperactivity symptoms by the Rutter teacher and parent questionnaires, revealed that genetic or congenital factors contribute to ~75% of the variance in attention and hyperactivity challenges (Goodman and Stevenson, 1989).

In the late 1980s and 1990s, public and scientific views on ADHD and stimulant medication like Ritalin shifted dramatically. Among increased usage in schools, serious concerns about over-prescription and side effects, such as violence and emotional disturbance, grew. Despite media reports and widespread public skepticism, the medical community continued with these drug treatments as part of a comprehensive ADHD strategy. This controversy led to hesitancy among parents and legal challenges, impacting ADHD treatment perceptions and practices significantly (Barkley, 2015).

### 6.4 Advances in behavioral and pharmacological treatment

In the 1980s, ADHD treatment advanced through the development and evaluation of behavioral parent training, cognitive-behavioral therapy (CBT), and increasingly sophisticated classroom management techniques. Social skills training also became increasingly investigated as a behavioral intervention for children with ADHD, although subsequent research raised concerns regarding the generalization and long-term effectiveness of treatment gains (Pfiffner and McBurnett, 1997).

In 1981, Firestone et al. evaluated behavioral parent training in hyperactive children, either alone or in combination with methylphenidate. Although all treatment groups showed improvements in home and school behavior, medication produced greater gains in attention, impulse control, academic achievement, and classroom behavior, and the addition of parent training did not provide significant benefit beyond medication alone (Firestone et al., 1981).

In 1982, Kendall and Braswell published one of the foundational studies in the history of CBT for hyperactive children, demonstrating its effectiveness. The children in their study were described as exhibiting “non-self-controlled behavior that interfered with academic and social performance,” matching classic hyperactivity/inattention profiles (Kendall and Braswell, 1982).

In 1985, Brown et al. further evaluated cognitive-behavioral approaches for hyperactive children and found that, although CBT was theoretically aimed at improving self-directed speech, problem-solving, and behavioral control, its effectiveness in clinical settings was limited (Brown et al., 1985).

In 1987, Pfiffner et al. reported that classroom management techniques, used either as alternatives or adjuncts to medication, were associated with improvements in academic achievement (Pfiffner and O'Leary, 1987).

Pharmacological treatment also expanded during the 1980s. In 1985, Hunt et al. reported the use of clonidine in selected ADHD cases, and subsequent studies further examined its potential therapeutic role (Hunt et al., 1985, 1990). Tricyclic antidepressants were also investigated during this period (Biederman et al., 1986, 1989).

Overall, the 1980s transformed ADHD into a more clearly defined and empirically investigated disorder. Refinements in diagnostic criteria, early neuroimaging findings, emerging genetic and motivational models, and broader behavioral and pharmacological interventions shifted the field toward a more differentiated understanding of ADHD as a clinically and biologically heterogeneous condition.

## 7 The 1990s

In 1990, Public Law 94–142 was reauthorized as the Individuals with Disabilities Education Act (IDEA). This reauthorization, later in the 1990s, led to the inclusion of children with ADHD in the “Other Health Impaired” category. This change allowed children with ADHD to access special educational services if their symptoms significantly impaired their academic performance (Barkley, 2015).

### 7.1 Neuroimaging research

In the early 1990s, there was a well-founded suspicion that delayed or impaired brain functioning, particularly in the frontal lobes, might play a role in this disorder. Throughout the 1990s, breakthroughs in neuroimaging technology provided new, insightful developments in identifying the structural and functional brain differences associated with this disorder (Giedd et al., 2001). Numerous neuropsychological studies during this period revealed that children with ADHD showed deficits in tasks presumed to assess frontal lobe or executive functions (Barkley et al., 1992).

The neuroimaging era of ADHD research advanced substantially in 1990, when Zametkin et al. used positron emission tomography (PET) with [^1^8F]FDG to assess brain metabolic activity in adults with ADHD. The study showed that global cerebral glucose metabolism was 8.1% lower in the ADHD group than in the control individuals. Particularly, the frontal and striatal regions exhibited the most significant reductions in metabolic activity (Zametkin et al., 1990). These findings aligned with earlier reduced CBF observations in these regions in children with ADHD (Lou et al., 1984, 1989). Zametkin et al.'s study was significant for its well-defined ADHD adult sample, thereby standing as one of the most explicit demonstrations of diminished brain activity in ADHD, particularly in frontal regions.

In 1990, Hynd et al. conducted one of the pioneering magnetic resonance imaging (MRI) studies examining cerebral structure in children with ADHD and found smaller anterior cortical regions, predominantly in the right hemisphere, along with an absence of the normal right-left asymmetry of the frontal cortex (Hynd et al., 1990).

In 1991, Hynd et al. (1991) found reductions in the size of both the anterior and posterior sections of the corpus callosum; however, subsequent research on the size of the corpus callosum provided somewhat inconsistent findings. In 1994, Semrud-Clikeman et al. noted a significantly smaller size in only the posterior region of the corpus callosum (Semrud-Clikeman et al., 1994), and a separate study in 1994 by Giedd et al. (1994) revealed marked reductions in the anterior portions, notably the rostrum and rostral body. In 1993, Hynd et al. (1993) also noted a smaller left caudate region in children with ADHD.

Later, in 1996 and 1997, research identified a reduced size in the prefrontal-caudate network among children with ADHD. As a result, it wasn't until the 1990s that adult psychiatry widely recognized the adult manifestation of childhood ADHD and began endorsing stimulant or antidepressant treatments (Spencer et al., 1995).

In the mid-1990s, despite some inconsistencies across MRI studies and considering the findings of Filipek et al. (1997), who observed the left striatal region to be smaller than the right (Filipek et al., 1997), most studies with considerably larger samples of children with ADHD demonstrated that the prefrontal-striatal regions were smaller in these children, with the right prefrontal region being smaller than the left (Castellanos et al., 1994, 1996). These results support the perspective that ADHD is indeed associated with developmental impairments in the brain, especially within the prefrontal-striatal network, and suggest that these deficits likely emerge early in neurodevelopment.

Functional neuroimaging research followed in 1998, when Vaidya et al. conducted the first fMRI study in ADHD, examining 10 male children with ADHD and 10 male controls during two versions of the go/no-go task. Response inhibition was impaired in children with ADHD on both tasks. Imaging results revealed that ADHD is characterized by atypical fronto-striatal function. Methylphenidate increased activation in frontal and striatal regions and improved response inhibition across both task conditions (Vaidya et al., 1998).

### 7.2 Emergence of seminal pathophysiological models of ADHD

In 1990, Joseph Sergeant proposed the “Cognitive-Energetic Model” or “State Regulation Deficit Model” for ADHD, based primarily on deficiencies in information processing (Sergeant and van der Meere, 1990). The original version of this model addressed ADHD deficiencies at two distinct levels (Sergeant and Van Der Meere, 1994). The model attempted to explain ADHD symptomatology through both “process” deficits and “energetic” factors, which include arousal, activation, and effort. The model suggested that in ADHD, one or more of the energetic pools (e.g., arousal, activation, or effort) may be defective, preventing an individual from recruiting and sustaining energy or effort to maintain performance. The updated version of this model was further developed to include “cognitive strategies” in cognitive processes (Sergeant, 2000).

A major executive-function account emerged in 1997, when Barkley proposed that ADHD symptoms result from deficits in behavioral inhibition and executive functioning, primarily stemming from the brain's inability to inhibit or delay behavioral responses. In formulating his original “Behavioral Inhibition Model” (Barkley, 1997), Barkley built upon the prior work of Quay (Quay, 1988) and Gray (Gray, 1982), positioning deficient behavioral inhibition as the central deficit in ADHD. This construct was conceptualized as comprising three interrelated processes: inhibition of the initial response, termination of an ongoing response, and interference control. In 1997, in his book titled “ADHD and the Nature of Self-Control,” Barkley explicitly stated that his inhibition model primarily applies to the hyperactive-impulsive and combined presentations, and not the inattentive presentation. He further noted that symptoms such as daydreaming, frequent staring, being in a fog, and being easily confused, as well as hypoactive and passive behaviors commonly observed in the inattentive presentation, seem to be related to a deficit in the speed of information processing generally, and focused or selective attention specifically (Barkley, 1997a).

### 7.3 Introduction of DSM-IV and the expanding international recognition of ADHD

In 1994, the DSM-IV introduced updated diagnostic criteria for the disorder (American Psychiatric Association, 1994), offering several advancements over the DSM-III-R's criteria. The DSM-IV introduced three subtypes of ADHD: predominantly inattentive type, predominantly hyperactive-impulsive type, and combined type. These subtypes reflect the different symptom patterns that people with ADHD may display. Additionally, the new criteria required evidence of the symptoms' widespread presence across various settings, along with proof of significant impairment in key areas of daily life functioning, such as at home, in school, or at work. The DSM-IV's criteria for ADHD, grounded in a far more extensive field trial than those of its predecessors, represented the most evidence-based standards for diagnosing ADHD up to that point.

Alongside these diagnostic developments, the late 1990s saw the rise of trends that would continue to evolve and expand throughout the following decade. This included the expanding recognition and treatment of the disorder in countries outside the U.S. and Canada, such as Portugal (Fonseca et al., 1995), Israel (Shalev et al., 1995), England (Toone and Van Der Linden, 1997), and France (Vermeersch and Fombonne, 1995).

### 7.4 Basic genetic research

#### 7.4.1 Twin and family studies

During the 1990s, converging family and twin studies substantially strengthened earlier evidence that ADHD aggregates within families and has a strong genetic component (Morrison and Stewart, 1971; Willerman, 1973). Biederman et al. reported that up to 35% of the immediate family members of children with ADHD were also diagnosed with the disorder (Biederman et al., 1990, 1992), and later found that offspring of a parent with ADHD had an estimated ~57% risk of the disorder (Biederman et al., 1995). Complementing these family findings, Gilger et al. reported that when one twin had ADHD, the likelihood of ADHD in the co-twin was 81% for monozygotic twins compared with 29% for dizygotic twins (Gilger et al., 1992). Subsequent large-scale twin studies further reinforced this evidence, estimating that genetic factors accounted for approximately 70–90% of the variance in hyperactivity–impulsivity symptoms, with an average contribution of ~80% (Gjone et al., 1996; Rhee et al., 1999). Collectively, these findings helped establish heritability as a central component of the etiological understanding of ADHD.

#### 7.4.2 Molecular genetics studies

During the 1990s, molecular genetic research extended the growing evidence for ADHD heritability by beginning to investigate specific candidate genes, particularly those related to dopaminergic neurotransmission. Early work implicated the dopamine receptor D2 (DRD2) gene, with Comings et al. reporting a higher prevalence of the A1 allele among children with ADHD than controls (Comings et al., 1991). Subsequent family-based studies identified an association between ADHD and the 10-repeat allele of the dopamine transporter gene (DAT1) (Cook Jr. et al., 1995), which was later replicated using similar clinical and laboratory methods (Gill et al., 1997). LaHoste et al. (1996) further reported an overrepresentation of the seven-repeat variant of the dopamine receptor D4 (DRD4) gene in children with ADHD. Collectively, these early candidate-gene studies shifted genetic research from demonstrating familial and heritable influences toward investigating specific molecular pathways that might contribute to ADHD susceptibility.

### 7.5 Recognition and developmental continuity of ADHD in adolescence and adulthood

Literature on the persistence of childhood hyperactivity into adulthood emerged in the late 1960s and 1970s, beginning with (Menkes et al., 1967), f and followed by longitudinal studies from Mendelson et al. (1971), Stewart and Morrison (1973), and Wood et al. (1976). Nevertheless, adult ADHD was not widely recognized as a valid disorder until the 1990s, and ADHD continued to be viewed predominantly as a transient childhood condition expected to resolve by puberty (Munoz-Millan and Casteel, 1989).

In 1996, Hill and Schoener reinforced this view by modeling persistence rates from studies following children with ADHD into adolescence or adulthood (Hill and Schoener, 1996). They predicted an exponential decline in ADHD rates, estimating adult ADHD prevalence at approximately 0.8% at age 20 and 0.05% at age 40. Their conclusion that ADHD essentially disappeared in adulthood was criticized by Russell Barkley a year later in

“*Age Dependent Decline in ADHD: True Recovery or Statistical Illusion?*” (Barkley, 1997b) and in his book “*ADHD and the nature of self-control*” (Barkley, 1997a).

In contrast, follow-up studies in the late 1980s and early 1990s showed that up to 70% of individuals continued to experience symptoms into adolescence and as many as 66% into adulthood (Safer and Krager, 1988; Barkley et al., 1990; Biederman et al., 1993).

In 1997, Seidman et al. examined whether neuropsychological impairments in ADHD persist into older adolescence. They found that both younger children and older adolescents with ADHD performed worse than age-matched controls on measures of attention and executive functioning. These findings demonstrated that neuropsychological impairments are not confined to younger children and challenged the view that such deficits simply reflect a maturational delay that resolves by adolescence (Seidman et al., 1997).

While initial evidence from smaller studies by Paul Wender and his team in the 1970s and 1980s had suggested the potential efficacy of psychostimulant drugs, namely pemoline (Wender et al., 1981) and methylphenidate (Wender et al., 1985), in treating ADHD in adults, it was more rigorous studies conducted in the mid-1990s that confirmed the effectiveness of methylphenidate (Spencer et al., 1995) and antidepressants such as desipramine (Wilens et al., 1996) in managing adult ADHD. Consequently, by the late 1990s, the prevailing view was that the adult manifestation of ADHD not only exhibited similar symptom patterns and comorbid disorders as its childhood counterpart but also showed comparable responsiveness to the same medications that had been effective in treating childhood ADHD.

### 7.6 Advancements in pharmacological and multimodal treatment in ADHD management

In 1996, the FDA approved Adderall, a mixed amphetamine salts formulation (amphetamine and dextroamphetamine), for the treatment of ADHD. In 1998, one of the early randomized controlled trials (RCTs) demonstrating the efficacy of Adderall in children with ADHD was published, involving a sample of 30 participants. Rapid enhancements in teacher ratings and children's math performance were observed by 1.5 h after Adderall administration, and these effects lasted until the end of the day. The study provided valuable insights into Adderall's effectiveness, making it a pivotal piece of literature for understanding the medication's role in managing ADHD symptoms in children (Swanson et al., 1998).

In 1997, another groundbreaking study, sponsored by the Swedish government, stood out for its extensive evaluation of stimulant medication in ADHD treatment, spanning a notable 15-month period (Gillberg et al., 1997). The findings supported sustained amphetamine efficacy in reducing inattention, hyperactivity, and related disruptive behaviors, with fewer treatment failures and a longer time to treatment failure in the amphetamine group. However, the study did not address potential adverse effects associated with longer-term amphetamine use.

In 1999, a major advance in research on the long-term efficacy of ADHD treatments came with the NIMH-funded Multimodal Treatment of ADHD (MTA) study, which provided one of the most comprehensive evaluations of pharmacological, behavioral, and combined treatment approaches (Group, 1999). The study compared the effects of pharmacotherapy, intensive behavior therapy, their combination, and standard community care over 14 months in children with ADHD combined type. For the majority of ADHD symptoms, significant enhancements were seen in children receiving either combined treatment or pharmacotherapy, surpassing the outcomes for those undergoing intensive behavioral therapy or provided with standard community care. Although combined treatment did not substantially outperform pharmacotherapy for core ADHD symptoms, it provided additional benefits across several behavioral, social, family, and academic outcomes. These findings supported pharmacotherapy as an effective treatment while highlighting the broader benefits of a multimodal approach.

In sum, the 1990s marked a major convergence of diagnostic, neuroimaging, genetic, neuropsychological, and treatment research in ADHD. Together, these advances strengthened the conceptualization of ADHD as a neurodevelopmental disorder with substantial biological and clinical heterogeneity, while growing recognition of its persistence into adulthood and the benefits of multimodal treatment expanded the disorder beyond its traditional framing as a childhood behavioral.

## 8 The 2000s to the present

Trends that emerged in the 1990s continued into the early 21st century, characterized by a substantial increase in neuroimaging research, the development of diverse pathophysiological models of ADHD, expanding evidence from family aggregation and heritability studies, growing investigation of the molecular genetic architecture of the disorder, and heightened interest in non-stimulant pharmacotherapies and non-invasive neuromodulation approaches.

### 8.1 Neuroimaging research

During the early 2000s, longitudinal neuroimaging increasingly supported a developmental rather than fixed-deficit account of ADHD. Castellanos et al. demonstrated developmental abnormalities in basal ganglia structures, particularly the caudate nucleus, with greater volume reductions in younger children, helping establish the importance of fronto-striatal circuitry in ADHD (Castellanos et al., 2002). Subsequent longitudinal studies by Shaw et al. (2006) extended this perspective to cortical development, showing reduced prefrontal cortical thickness associated with poorer clinical outcomes and, importantly, delayed attainment of peak cortical thickness in children with ADHD compared with typically developing peers (Shaw et al., 2007). Together, these findings strengthened the conceptualization of ADHD as a disorder involving altered neurodevelopmental trajectories rather than simply static structural abnormalities.

Subsequent large-scale and meta-analytic research broadened this neurodevelopmental framework from individual regions and circuits toward distributed brain systems. A meta-analysis of 55 task-based fMRI studies demonstrated abnormalities across multiple large-scale networks, including fronto-parietal, ventral attention, default mode, and sensorimotor networks, helping shift the field toward a systems-level perspective (Cortese et al., 2012). This broader view was reinforced by the ENIGMA ADHD mega-analysis of more than 3,200 individuals, which identified smaller volumes across several subcortical regions, with the strongest differences observed in childhood (Hoogman et al., 2017). More recently, a meta-analysis of 129 diffusion imaging studies identified white-matter microstructural differences across projection, commissural, and association pathways, particularly the corpus callosum and cingulum, further emphasizing large-scale connectivity abnormalities in contemporary models of ADHD (Parlatini et al., 2023).

### 8.2 Emergence of new pathophysiological models of ADHD

In 2002, Sonuga-Barke proposed a “Dual-Pathway Model” defining ADHD as the product of two distinct processes or pathways, one mediated by impaired inhibitory control and the other underpinned by delay aversion (Sonuga-Barke, 2002). Indeed, Sonuga-Barke built his dual-pathway model based on the findings of a “head-to-head” NIMH Multimodal Treatment study (Solanto et al., 2001) which showed that delay aversion and poor inhibitory control in ADHD are dissociated from each other and are independent co-existing characteristics of the combined presentation of ADHD. According to the dual-pathway model, poor inhibitory control is a characteristic of the dysregulation of action and thought, which is associated with the meso-cortical branch of the dopamine system projecting to cortical cognitive control centers (e.g., PFC), while delay aversion is thought to underpin ADHD individuals' motivational style, which is associated with the meso-limbic dopamine branch related to the reward circuits (e.g., nucleus accumbens) (Sonuga-Barke, 2003).

In 2003, El-Sayed et al. proposed the “Maturational Lag Hypothesis,” suggesting that the disorder reflects a delay in central nervous system development rather than a deviation from typical developmental trajectories. Within this framework, ADHD symptoms are conceptualized as arising from delayed maturation, whereby children exhibit neurodevelopmental patterns resembling those of younger typically developing individuals and may partially normalize with age. The extent of this “catch-up” is thought to depend, in part, on the severity and persistence of initial symptoms (El-Sayed et al., 2003). Nevertheless, the model has been widely debated, particularly in light of competing evidence suggesting that ADHD may reflect atypical developmental trajectories rather than a simple maturational delay (Chabot and Serfontein, 1996).

By the early 2000s, the field was increasingly moving beyond single-deficit explanations toward models incorporating multiple cognitive, executive, motivational, and developmental pathways. In 2005, Sonuga-Barke published a review article highlighting the need to consider multiple developmental pathways models rather than a simple causal model of single, common core dysfunctions in ADHD. He emphasized the role of the child's social environment in shaping neurodevelopmental pathways to ADHD. Based on evidence of neuropsychological heterogeneity in ADHD, he argued for the likelihood that multiple neurodevelopmental pathways underpin ADHD and emphasized the need for theoretical models of ADHD to integrate both cognitive (e.g., inhibitory-based executive processes) and motivational (delay-related motivational processes) elements (Sonuga-Barke, 2005). Sonuga-Barke's insight opposed single-core deficit models, which indicate that because ADHD is a unitary construct at the level of clinical symptoms, it requires definitions in terms of a single core deficit facilitated by a common set of psychopathophysiological pathways (Barkley, 1997).

In 2005, Douglas proposed a “Self-Regulation Model,” conceptualizing the disorder as reflecting impairments across three partially dissociable components, namely, effortful attention, inhibitory control, and strategic/organizational processes. Douglas emphasized the importance of distinguishing among these components to avoid misattributing performance failures and argued that ADHD heterogeneity may arise from different combinations of self-regulatory deficits, thereby reinforcing the need for component-specific assessment and theory development (Douglas, 2005).

In 2005, Nigg and Casey argued that a cohesive understanding of ADHD mandates integrating an account of both cognitive control systems and affective-response neural systems involved in detecting the emotional significance of events (Nigg and Casey, 2005). According to their “Integrated Affective and Cognitive Model,” the neural circuitries relevant to ADHD consist of: (a) fronto-striatal (i.e., cortico-basal ganglia-thalamo) circuitries involved in response inhibition, response selection, and working memory; (b) fronto-neocerebellar circuitries involved in temporal information processing; and (c) fronto-limbic circuitries involved in reinforcement learning and avoidance-related behavior. In view of this model, impulsivity seems to result from an unregulated approach to all potential rewards, as well as a failure to avoid potential punishment (Nigg and Casey, 2005).

In 2005, Sagvolden et al. proposed the “Dynamic Developmental Theory,” hypothesizing that two key behavioral processes might underlie ADHD symptoms, altered reinforcement of novel behavior and deficient extinction of previously reinforced behavior (Sagvolden et al., 2005). This theory attributed these alterations to hypofunction of the mesolimbic dopamine system. Impairment of the mesocortical dopamine pathway is associated with deficits in attention and behavioral organization, whereas dysfunction of the nigrostriatal dopamine pathway disrupts motor function and non-declarative habit learning. By incorporating molecular principles from the “tonic/phasic model of dopamine system regulation” proposed by Grace (1991), the dynamic developmental theory posits that ADHD involves dysregulation of tonic and phasic dopamine signaling. Specifically, reduced tonic dopamine levels are thought to diminish the gain of phasic dopamine responses, resulting in attenuated phasic signaling to salient stimuli. Within this framework, decreased dopaminergic activity in fronto-limbic circuitry contributes to weaker reward-related phasic responses, delayed shifts in dopamine signaling, and reduced sensitivity to reward-predictive cues (Sagvolden et al., 2005).

In 2006, Halperin and Schulz argued that although most of the neuropathophysiology models of ADHD have focused on the PFC and its interconnections with subcortical structures, executive dysfunctions in ADHD appear early in childhood, are unrelated to cortical neural dysfunctions, and tend to recover as the child develops. They proposed that, while executive deficits are common in ADHD, the disorder's cognitive impairments may instead reflect primary deficits in more basic sensory-motor, perceptual, and linguistic processes that executive functions serve to integrate. This perspective led to the introduction of a “Neurodevelopmental Model” of ADHD, positing that childhood ADHD is apparently due to non-cortical neural dysfunctions, such as those in the basal ganglia, locus coeruleus, and cerebellum, which are present early in ontogeny and remain relatively static throughout an individual's lifespan (Halperin and Schulz, 2006).

Network-based conceptualizations gained further prominence in 2007 with Sonuga-Barke and Castellanos's “Default Mode Network (DMN) Interference Hypothesis,” emphasizing attentional dysfunction as a core impairment. According to this model, deficits in sustained attention arise from a failure to appropriately transition between, or maintain the transition from, a resting to a task-engaged cognitive state. This failure is attributed to interference from task-irrelevant activity within the DMN, which disrupts the functioning of task-active networks, including the frontoparietal (executive control) and ventral attention (salience) networks. From this perspective, increased reaction time variability in ADHD is thought to reflect intermittent attentional lapses arising from the spontaneous and random emergence of DMN activity during task performance, thereby compromising attentional control. Overall, this model conceptualizes ADHD as a disorder of dysfunctional large-scale network connectivity (Sonuga-Barke and Castellanos, 2007).

In 2008, Tripp and Wickens proposed the “Dopamine Transfer Deficit Theory,” which posits altered sensitivity to positive reinforcement in ADHD, driven by a dysfunction in dopamine transfer (Tripp and Wickens, 2008). According to this model, the normal transfer of phasic dopamine neuron responses from primary reinforcement to previously neutral predictive cues is disrupted. As a result, the anticipatory dopamine signal is attenuated, leading to ineffective reinforcement at the cellular level. This impairment reduces the influence of predictive cues and enhances responsiveness to immediate reinforcement, as phasic dopamine activity remains relatively tied to the actual delivery of rewards rather than their anticipation. Consequently, individuals with ADHD exhibit heightened sensitivity to local or recent reinforcement contingencies and diminished sensitivity to broader, global contingencies. Within this framework, inattentiveness and impulsivity are understood as behavioral consequences of this imbalance, reflecting a bias toward immediate environmental feedback over internally represented reinforcement structures (Tripp and Wickens, 2008).

In 2010, Sonuga-Barke conducted a clinical trial and used principal component factor analysis to test whether temporal processing-related impairments represent a third independent neuropsychological component, alongside the inhibitory control and delay-related deficits of the dual-pathway model of ADHD. It appears they became interested in testing this hypothesis based on MRI findings, which showed that, although temporal processing may share neural components with the inhibitory system of the dorsal fronto-striatal circuit and the delay aversion system of the ventral fronto-striatal circuit, the cerebellum is exclusively involved in temporal processing. The findings from this study laid the foundation for the “Triple-Pathway Model” of ADHD, demonstrating that temporal processing (timing) deficits represent a distinct third neuropsychological pathway. This model, for the first time, showed that three separable components of inhibition, delay aversion (reward), and timing contributed to the variance in their neuropsychological task battery, suggesting that deficits in these domains in ADHD are dissociable from each other, with large subgroups of individuals affected in only one domain (Sonuga-Barke et al., 2010).

In 2011, in support of Sonuga-Barke's triple-pathway model, Durston et al. also aimed to address neuropsychological heterogeneity in ADHD by investigating whether affected neurocognitive functions and their associated neurobiological systems are separable. They proposed a neurobiological framework for ADHD, similar to Sonuga-Barke's model, suggesting three distinct pathways: cognitive control (dorsal fronto-striatal), reward and motivation (ventral fronto-striatal or orbitofronto-striatal), and timing (fronto-neocerebellar). They also proposed, for the first time, the existence of a fourth component involving deficits in vigilance (Durston et al., 2011).

In 2015, Rebeca Heidbreder set forth the idea that ADHD symptomatology is best conceptualized as a continuum, suggesting that the construct of a spectrum could better handle heterogeneity (e.g., symptom presentation, neuropsychological assessment, comorbidity, etc.) and individual variability among people with ADHD (Heidbreder, 2015). Heidbreder further expanded on this idea by borrowing the concept of the “Attentional Spotlight” from Shalev and Tsal (2003), proposing that while both the hyperactive-impulsive and combined presentations may have too wide attentional spotlights, the inattentive presentation may have a too narrow attentional spotlight. This suggests that the presentations may vary as a function of the breadth of the attentional spotlight.

In 2017, building on earlier conceptualizations of ADHD as a spectrum, the Hierarchical Taxonomy of Psychopathology (HiTOP) extended this dimensional perspective by embedding ADHD within a broader, hierarchically organized structure of psychopathology. Rather than conceptualizing ADHD solely along a symptom continuum, HiTOP situates it within the externalizing spectrum, alongside related conditions such as oppositional defiant disorder and conduct disorder, reflecting shared liabilities including impulsivity and behavioral dysregulation. This framework emphasizes that ADHD symptoms exist along continuous dimensions and frequently co-occur with other forms of psychopathology, thereby challenging rigid diagnostic boundaries (Kotov et al., 2017). HiTOP integrates findings from genetics, neurocognition, and neuroimaging, which consistently demonstrate overlapping mechanisms across psychiatric conditions. Within this framework, ADHD is no longer viewed as an isolated disorder but as part of a broader network of dimensional traits, providing a more comprehensive account of its heterogeneity, comorbidity, and developmental variability (Kotov et al., 2021).

### 8.3 Introduction of DSM-IV-TR, DSM-5, and DSM-5-TR

In 2000, the DSM-IV-TR (Dsm-Iv-tr, 2000) was introduced, and it did not change the criteria for diagnosing ADHD compared to the DSM-IV. Since the year 2000, there has been a notable escalation in research aimed at subclassifying ADHD. The emerging evidence from these studies casts doubts on the efficacy of the DSM-IV's subtyping framework for ADHD, suggesting that ADHD may instead be a singular condition with variable severity among individuals and that it comprises two symptom dimensions that, although highly interconnected, are also somewhat distinct. In addition, researchers and clinicians realized that the traditional inattentive, hyperactive-impulsive, and combined subtypes of ADHD are developmentally unstable. For instance, an individual can meet the criteria for the hyperactive-impulsive subtype during early childhood (ages 3–6), the combined subtype during middle childhood and early adolescence (ages 7–12), and the inattentive subtype as a young adult (ages 18–25) (Asherson and Network, 2013; Curchack-Lichtin et al., 2014).

In 2012, because of the instability of the clinical subtypes, the committee of the DSM-5, proposed eliminating ADHD subtypes and instead suggested the concept of “presentations” (Barkley, 2013a,b). In 2013, the DSM-5 (APA, 2013) retained the three subtypes of ADHD from the DSM-IV but referred to them as “presentations” instead of “subtypes” to reflect the fluidity of symptoms over time. Rather than describing ADHD as separate subtypes, the DSM-5 acknowledges ADHD as a single disorder that can manifest differently in individuals, with a variety of symptoms on a spectrum. This transition reflects a more nuanced understanding of the disorder, recognizing that symptoms can change over time and that an individual may not fit neatly into one category (Curchack-Lichtin et al., 2014). In 2022, the DSM-5-TR was introduced, building upon the foundation laid by the DSM-5 without altering the diagnostic criteria for ADHD. Instead, it aimed to refine the understanding and classification of the disorder by incorporating the latest research and clinical insights. This edition emphasized recognizing ADHD as a dynamic condition, with symptom presentations that can evolve across an individual's lifespan, thereby supporting a more nuanced approach to diagnosis and treatment planning (American Psychiatric Association, 2022).

### 8.4 Genetic research

In the first decade of the 21st century, many potential genes associated with the disorder were discovered, alongside novel chromosomal areas warranting further exploration through comprehensive scans of the human genome. This field of study has shown that ADHD is influenced not only by genes regulating dopamine and norepinephrine networks but also by other genes that play roles in neuronal development, the termination of growth endpoints, and the sprouting of neurons (Faraone et al., 2005; Stergiakouli and Thapar, 2010).

In 2000, the first study to examine the association between the serotonin 2A receptor gene (HTR2A) and ADHD reported preferential transmission of the T allele of rs6314 to ADHD probands, although no association was observed for rs6313 (Quist et al., 2000).

In 2001, the first study to examine linkage and association between the nicotinic acetylcholine receptor α4 subunit gene (CHRNA4) and ADHD reported no evidence of association with a polymorphism in exon 5 using a transmission disequilibrium test in a sample of 70 children (Kent et al., 2001). Similarly, Xu et al. conducted the first study of the adrenergic α2A receptor gene (ADRA2A) in ADHD and found no evidence of association with a promoter-region polymorphism (rs1800544) using a transmission disequilibrium test in a sample of 94 families (Xu et al., 2001). In contrast, Manor et al. (2001), in the first study of the serotonin transporter-linked polymorphic region (5-HTTLPR) in ADHD, reported an over-representation of the long allele among children with the combined subtype in a parent–proband trio sample. Collectively, these early candidate gene studies yielded mixed findings, underscoring the complexity and heterogeneity of ADHD genetics and motivating the transition toward larger-scale genome-wide approaches.

In 2008, the first genome-wide association study of ADHD, conducted in 909 parent–child trios from the International Multicenter ADHD Genetics (IMAGE) study, did not identify any genome-wide significant associations for the categorical diagnosis (Neale et al., 2008). However, secondary analyses of ADHD-related quantitative traits revealed genome-wide significant associations for variants in the CDH13 gene, encoding the neural adhesion protein cadherin 13, and in the GFOD1 gene, which encodes a protein of unknown function (Neale et al., 2008).

In 2010, one of the first meta-analyses of genome-wide association studies (GWAS) of childhood ADHD, conducted by Neale et al., combined data from multiple cohorts to increase statistical power for detecting common genetic variants. Despite this large-scale effort, no genome-wide significant loci were identified. However, several loci demonstrated suggestive associations, although none were consistently replicated across datasets. These findings highlighted the absence of single variants with large effects and instead pointed to a highly polygenic architecture, in which numerous variants of small effect contribute to ADHD risk. The study also underscored substantial genetic heterogeneity and the limitations of early GWAS approaches, particularly those with relatively modest sample sizes (Neale et al., 2010). Overall, these results reinforced the notion that ADHD cannot be explained by individual candidate genes alone and motivated the need for larger consortia and more refined, dimensionally informed genetic analyses.

In 2013, a landmark genome-wide single-nucleotide polymorphism (SNP)-based analysis by the Psychiatric Genomics Consortium provided one of the first molecular estimates of heritability across five major psychiatric disorders, including ADHD. This study reported a SNP-based heritability (${\text{h}}_{\text{snp}}^{2}$) of ~28% for ADHD, affirming that common genetic variants substantially contribute to its etiology. Importantly, the study also identified a moderate genetic correlation (r_snp_ = 0.32) between ADHD and major depression, suggesting overlapping polygenic influences. These findings marked a pivotal shift toward quantifying shared genetic liability between psychiatric conditions using molecular data (Devlin et al., 2013).

In 2018, ADHD Working Group of the Psychiatric Genomics Consortium conducted the first genome-wide association study to identify genome-wide significant risk loci for ADHD, marking a major breakthrough in the field. Analyzing data from 55,374 individuals, the study identified 12 independent loci significantly associated with ADHD risk. The estimated SNP-based heritability was ~22%, confirming the polygenic nature of ADHD. These findings provided robust evidence for specific common genetic variants contributing to ADHD susceptibility and opened new pathways for biological understanding and pharmacotherapy discoveries (Demontis et al., 2018).

In 2023, the largest genome-wide association study to date on ADHD, encompassing 38,691 ADHD cases and 186,843 controls, identified 27 genome-wide significant loci, including 21 novel ones. This study confirmed that ADHD is highly polygenic, influenced by around 7,300 common variants, and revealed a striking genetic overlap (84–98%) with other psychiatric disorders such as autism spectrum disorder (ASD) and major depression. It also found that common ADHD risk variants are enriched in genes expressed in the frontal cortex and midbrain dopaminergic neurons. Importantly, polygenic ADHD risk was associated with impairments in attention, verbal reasoning, and working memory, further linking genetic risk to core cognitive symptoms of ADHD (Demontis et al., 2023).

### 8.5 Advances in adult ADHD

In the early 2000s, accumulating evidence supporting the validity and prevalence of adult ADHD, along with limited awareness in primary care settings, underscored the need to establish accurate diagnostic approaches and appropriate models of care for adults with ADHD in both psychiatric and primary care settings.

In 2000, Faraone et al. published a comprehensive overview of adult ADHD, demonstrating its validity as a diagnosis by showing comparable patterns of psychiatric comorbidity, functional impairment, medication response, molecular genetic features, and neuropsychological profiles to those observed in children and adolescents with ADHD (Faraone et al., 2000).

In 2004, McGough and Barkley critically examined the application of DSM-IV criteria to adult ADHD, highlighting several limitations. They noted that DSM-IV symptoms were developed based on child populations and lack developmental appropriateness for adults, with some items lacking face validity (e.g., “runs and climbs excessively” and “has difficulty playing quietly”). Additionally, the diagnostic threshold of six symptoms and the age-of-onset criterion lack empirical support in adult populations, potentially leading to underdiagnosis. The authors emphasized that DSM-IV criteria have not been formally validated in adults and may fail to capture clinically significant impairment in this group (McGough and Barkley, 2004).

In 2004, Faraone published a review synthesizing the evidence on the genetics of adult ADHD up to that time, emphasizing strong evidence for its familial and genetic basis. He highlighted substantial familial aggregation, supporting the validity of adult ADHD as a diagnosis. He further suggested that persistent ADHD is more strongly familial than remitting forms, indicating a greater genetic contribution to symptom persistence (Faraone, 2004).

In 2015, Moffitt et al. challenged the long-standing assumption that adult ADHD is necessarily a continuation of childhood-onset neurodevelopmental disorder. Using a prospective four-decade birth cohort, they reported that the majority of adults diagnosed with ADHD at age 38 did not have a childhood history of the disorder. Moreover, these individuals lacked the neuropsychological deficits and genetic risk patterns typically associated with childhood-onset ADHD. Moffitt et al. suggested that late-onset adult ADHD may represent a distinct clinical entity, proposing that adult ADHD is not a neurodevelopmental disorder Moffitt et al., (2015).

In 2015, F. Xavier Castellanos (Castellanos, 2015) published an editorial emphasizing the urgent need for replication studies to confirm or challenge the findings of (Moffitt et al. 2015).

In 2016, in direct response to Castellanos' call for replication, Caye et al. conducted a population-based longitudinal study of 18–19-year-old young adults with ADHD to examine whether adult ADHD is always preceded by childhood-onset symptoms. They found that 87% of adult ADHD cases lacked a history of childhood ADHD, replicating and extending the findings of Moffitt et al. in a younger population. Based on these results, Caye et al. proposed the existence of two syndromes of adult ADHD with potentially distinct developmental trajectories: one early-onset and the other late-onset, with the latter appearing more prevalent in adulthood. Despite these differences, they suggested that late-onset ADHD could still be conceptualized as a neurodevelopmental condition (Caye et al., 2016).

In 2016, Agnew-Blais et al. further examined the developmental course of ADHD using a population-based longitudinal twin cohort. They reported substantial heterogeneity in adult ADHD, identifying three trajectories: persistent, remitted, and late-onset ADHD. Notably, among individuals meeting criteria for ADHD in young adulthood, approximately two-thirds did not have a history of childhood ADHD, reinforcing the presence of a large late-onset group. Compared with persistent ADHD, late-onset cases showed fewer childhood behavioral problems and higher childhood IQ, yet exhibited comparable levels of impairment and psychiatric comorbidity in adulthood. These findings supported the notion that adult ADHD is not a homogeneous continuation of childhood disorder and highlighted the importance of developmental heterogeneity in its conceptualization (Agnew-Blais et al., 2016).

In 2016, Faraone and Biederman, published an editorial synthesizing findings from longitudinal cohort studies conducted in New Zealand (Moffitt et al., 2015), Brazil (Caye et al., 2016), and the United Kingdom (Agnew-Blais et al., 2016). They acknowledged that these studies collectively suggested the possibility of adult-onset ADHD and even proposed that childhood-onset and adult-onset ADHD might represent distinct syndromes. However, they urged caution in interpreting these findings, highlighting potential methodological limitations, including reliance on adult self-reports and the risk of false-positive diagnoses. Importantly, Faraone and Biederman argued that many so-called adult-onset cases may reflect subthreshold or undetected childhood ADHD, rather than truly *de novo* onset in adulthood. From this perspective, they proposed a more parsimonious model in which ADHD reflects a dimensional and developmental disorder with variable timing of symptom emergence, rather than two entirely distinct syndromes (Faraone and Biederman, 2016).

In 2022, Taylor et al. conducted a systematic review critically evaluating the emerging literature on adult-onset ADHD. They concluded that the existing evidence was methodologically insufficient to support adult-onset ADHD as a valid, distinct clinical entity. Across studies, substantial heterogeneity in diagnostic procedures, reliance on retrospective self-report, and inconsistent application of DSM criteria raised concerns about recall bias and false-positive diagnoses, particularly in population-based designs. Importantly, they emphasized that many cases labeled as adult-onset ADHD could be better explained by alternative mechanisms, including subthreshold childhood symptoms that became clinically significant later in life, environmental or developmental changes that unmasked underlying vulnerabilities, or phenocopies arising from other conditions (e.g., sleep apnea, substance use, trauma, etc.). Furthermore, they highlighted that symptom definitions derived from childhood ADHD may not adequately capture adult presentations, complicating diagnosis. Overall, Taylor et al. argued for a cautious interpretation of late-onset findings and reinforced a developmental, dimensional framework in which apparent adult-onset ADHD often reflects delayed expression, misclassification, or methodological limitations rather than true *de novo* onset (Taylor et al., 2022).

### 8.6 Emergence of Cognitive Disengagement Syndrome as a Distinct Condition from ADHD

In 1985, Ben Lahey et al. (1985) raised the possibility of a new attention disorder, describing symptoms like drowsiness, sluggishness, and daydreaming in the ADD–H group. The nomenclature of “sluggish cognitive tempo” (SCT) was first introduced by this group in their 1987 publications for the condition (Carlson et al., 1987; Lahey et al., 1987).

In 2013, Barkley proposed the term “Concentration Deficit Disorder” (CDD) to more accurately describe the condition and reduce confusion, as well as to address the negative connotations associated with the term SCT, which was perceived by the public as offensive (Barkley, 2013a,b, 2014). Since 2014, there has been growing research interest in SCT/CDD, marking a significant shift in the scientific community's efforts to understand and distinguish SCT/CDD from ADHD (Fredrick et al., 2023).

In 2022, an international Work Group of 13 leading SCT experts convened to advance SCT research and agree on a new nomenclature. After repeated discussions, “Cognitive Disengagement Syndrome” (CDS) was chosen to replace “SCT” as the preferred term for the construct because it is more accurate and less demeaning than “SCT” (Becker et al., 2023).

### 8.7 Global recognition and the emergence of consensus in ADHD

Despite growing recognition of ADHD during the 1990s, the disorder remained controversial, particularly with respect to its validity, diagnostic boundaries, and the increasing use of stimulant medications by the early 2000s. These ongoing debates, coupled with variability in clinical practices and public skepticism, created a need for greater clarity and standardization. In response, a series of consensus statements were developed to synthesize the available evidence and guide clinical understanding.

In 1998, the National Institutes of Health (NIH) Consensus Statement on ADHD (NIH, 1998) represented one of the earliest formal efforts to address these issues, followed by the first International Consensus Statement on ADHD (Barkley, 2002), which sought to establish broader scientific agreement. This movement continued with the European Consensus Statement on Diagnosis and Treatment of Adult ADHD (Kooij et al., 2010), reflecting growing recognition of ADHD across the lifespan. Over time, these initiatives contributed to a gradual convergence toward a more unified international perspective, culminating in the World Federation of ADHD International Consensus Statement (Faraone et al., 2021), which involved 80 authors across 27 countries and was read and endorsed by 366 clinicians and researchers. This statement represents the most comprehensive and up-to-date globally aligned framework, comprising 208 statements for the understanding and management of ADHD.

In parallel, since the early 2000s, global recognition of ADHD has increased substantially, driven by the emergence of parent support groups, a notable rise in research output from both developing and non-Western countries, and the expansion of pharmacological treatment across a growing number of nations (Faraone et al., 2003; Hinshaw et al., 2011). The increasing accessibility of digital information platforms, including the Internet, has likely further facilitated the dissemination of knowledge about ADHD and contributed to a more uniform understanding of the disorder across regions.

### 8.8 Comorbidity in ADHD

In most individuals with ADHD, psychiatric and non-psychiatric comorbid disorders co-exist (Gillberg et al., 2004; Biederman et al., 2006). Historically, in the 1970s, American clinician and epidemiologist Alvan Feinstein introduced the term “co-morbidity” in clinical epidemiological studies to describe the presence of one or more additional ailments alongside the primary disease (Feinstein, 1970). The concept of “comorbidity” was first used in psychiatric literature in the mid-1980s (Barlow et al., 1986; McGlashan, 1987; Sanderson et al., 1990).

In 1991, Biederman et al. (1991) were the first to make a systematic search of the psychiatric and psychological literature for empirical studies dealing with the comorbidity of ADHD with other disorders, using the term “comorbidity.”

With the beginning of the 2000s, further work examined disorders that are likely to be comorbid with ADHD in both children and adolescents (Angold et al., 1999) and adults (Barkley et al., 2010). For example, the MTA study of children with ADHD (*N* = 579; ages 7–9.9 years) found that 69% of children fully met diagnostic criteria for one or more additional psychiatric disorders (Group, 1999).

In 2006, a notable epidemiological study also revealed that adults with ADHD are six times more likely to experience at least one additional psychiatric disorder during their lifetime compared to adults without ADHD (Kessler et al., 2006).

### 8.9 Long-term outcomes of ADHD treatment: findings from the MTA study

Several advances were also observed in the domain of treatment, for instance, with the continued reporting of results from the MTA (Group, 1999).

In 2004, the MTA reached another pivotal moment with the publication of its 24-month outcomes (Group, 2004). This follow-up study assessed the persistence of these beneficial effects 10 months beyond the 14 months of intensive therapeutic intervention, on a cohort of 540 children from the original 579 participants. The findings at the 24-month mark revealed a sustained, albeit reduced, superiority of the medication strategy over behavioral therapy and community comparison in managing ADHD and ODD symptoms. Contrary to the 14-month outcomes, no significant additional benefits were observed for the combination therapy over medication management alone, nor was behavioral therapy found to be superior to the community comparison. The study also highlighted the variance in medication doses across the groups, which partly mediated the ongoing superiority of the combined and medication management strategies. This 24-month follow-up report from the MTA study offered valuable insights into the long-term effects of the intensive treatment strategies for ADHD, revealing a diminishing yet persistent benefit of medication management in symptom management beyond the intensive treatment phase (Group, 2004).

In 2021, the MTA study examined the extent to which children with ADHD experience recovery in adulthood. Participants underwent eight assessments over follow-up periods ranging from 2 to 16 years after baseline. Only 9% of the sample demonstrated recovery (i.e., sustained remission) by the study endpoint, while approximately 64% exhibited fluctuating periods of remission and recurrence between childhood and young adulthood (Sibley et al., 2022). These findings challenged the widely held notion, based largely on single-point assessments, that approximately 50% of individuals with childhood ADHD outgrow the disorder by adulthood, highlighting the importance of examining longitudinal patterns of ADHD expression (Barkley et al., 2002; Sibley et al., 2017).

### 8.10 Development of non-stimulant ADHD medications

During the first decade of the 2000s, the FDA approved three new non-stimulant medications for managing ADHD, namely, Atomoxetine (Strattera), Guanfacine extended-release (Intuniv), and Clonidine extended-release (Kapvay).

In 2002, atomoxetine, a norepinephrine reuptake inhibitor, was approved by the FDA and marked the first non-stimulant medication for the management of ADHD in adults, children, and adolescents (Rosack, 2002). Distinguished by its lack of abuse potential, making it non-scheduled in the U.S., it offers an easier prescription alternative to Schedule II stimulants. Atomoxetine, extensively researched for FDA approval, is recommended as a second-choice treatment for ADHD in various professional guidelines, reflecting its significant role in ADHD management.

In 2009, the FDA approved guanfacine XR, the second non-stimulant for ADHD in children and adolescents aged 6 to 17 years, originally used for hypertension. Guanfacine XR acts similarly to clonidine as an alpha2 agonist, targeting impulsivity and emotional excitability. However, clonidine's cardiotoxicity risks and lack of specific FDA approval for ADHD limited its wider use, despite some combining it with stimulants for enhanced symptom management (Faraone et al., 2013).

In 2010, clonidine extended-release was approved by the FDA, making it the third non-stimulant treatment option for ADHD. Clonidine, an alpha-2 adrenergic agonist like guanfacine, had been utilized off-label for ADHD, particularly for its potential benefits in managing hyperactivity, impulsivity, and attentional challenges. With its FDA approval, Clonidine extended-release became an officially recognized treatment option, offering an alternative to those seeking non-stimulant medications for ADHD management (Jain et al., 2011).

### 8.11 Emergence of non-invasive brain neuromodulation techniques

#### 8.11.1 Neurofeedback

The earliest neurofeedback evidence emerged in 1976, when Lubar and Shouse reported EEG and behavioral changes in a hyperkinetic child following training of the sensorimotor rhythm (SMR, 12–14 Hz). Based on the association of SMR with reduced motor activity, they proposed that enhancing this rhythm could alleviate hyperactivity. Using an ABA design, they showed that symptoms decreased when SMR was increased and worsened when it was suppressed. This work laid the foundation for later neurofeedback protocols targeting EEG rhythms in ADHD (Lubar and Shouse, 1976).

In 2004, Heinrich et al. reported positive effects of slow cortical potential (SCP) neurofeedback in children with ADHD. Unlike frequency-based approaches, SCP training reinforced shifts in EEG polarity using a reward-based paradigm. The study showed improvements in core ADHD symptoms and suggested that both SCP and SMR neurofeedback may act through modulation of cortical excitability. These findings helped expand neurofeedback approaches and stimulated further research in ADHD (Heinrich et al., 2004).

Evidence from larger controlled studies followed in 2009, when Gevensleben et al. compared neurofeedback with computerized attention training in children with ADHD. Neurofeedback, including theta–beta and SCP protocols, led to significantly greater improvements in parent- and teacher-rated ADHD symptoms. This study provided one of the first robust demonstrations of neurofeedback efficacy beyond small preliminary trials (Gevensleben et al., 2009).

In 2013, Arnold et al. conducted a double-blind RCT to assess the feasibility of neurofeedback in children with ADHD and compare two vs. three sessions per week. Thirty-nine participants were enrolled with high completion rates and successful blinding. Both active NF and sham showed substantial symptom improvement, with no significant between-group differences. Three sessions per week were preferred but showed similar effectiveness to two sessions (Arnold et al., 2013).

In 2017, Strehl et al. performed a multicenter randomized trial comparing SCP neurofeedback with a semi-active control condition. Neurofeedback resulted in greater improvements in parent-rated ADHD symptoms, although differences in teacher ratings were less pronounced. The study strengthened evidence for neurofeedback while highlighting the importance of controlling for non-specific treatment effects (Strehl et al., 2017).

#### 8.11.2 Repetitive transcranial magnetic stimulation

Clinical investigation of rTMS in ADHD began in 2010 with Bloch et al.'s first single-blind crossover RCT in adults. A single session of 20 Hz rTMS was applied to the right dlPFC, and the self-reported Positive and Negative Affect Schedule (PANAS) were evaluated both before and 10 min after rTMS. The results showed an improvement in attention scores in the active rTMS group compared to the sham group, but no effect on hyperactivity, mood, or anxiety scores (Bloch et al., 2010).

The first multi-session evaluation of rTMS followed in 2012, when Weaver et al. studied adolescents and young adults with ADHD using a single-blind crossover RCT in nine participants. Participants received 10 sessions of 10 Hz rTMS over the right dlPFC across2weeks, including a sham condition. rTMS was safe, with no serious adverse events. Although overall clinical improvement was observed, no significant differences emerged between active and sham conditions (Weaver et al., 2012).

In 2014, Gómez et al. reported the first open-label study on rTMS in 10 children with ADHD. Participants received five consecutive daily sessions of 1 Hz rTMS over the left dlPFC. Significant improvements were observed in inattention (teacher-reported) and hyperactivity/impulsivity (parent-reported). However, the small sample size and lack of a control condition limited causal interpretation. The treatment was well-tolerated and appeared safe in this population (Gómez et al., 2014).

In 2018, Paz et al. conducted the first double-blind RCT examining bilateral rTMS over the dlPFC in adults with ADHD. Nine participants received 20 daily sessions of 18 Hz deep rTMS over 4 weeks. The intervention was safe and feasible but showed no significant between-group differences in clinical (CAARS) or cognitive (CPT, TOVA) outcomes. The authors attributed the lack of efficacy to the small sample size and the complexity of the stimulation parameters (Paz et al., 2018).

A larger comparative trial was reported in 2018, when Cao et al. evaluated rTMS, Atomoxetine, and their combination in children with ADHD. Participants received 30 sessions of 10 Hz rTMS over the right dlPFC, Atomoxetine, or combined treatment over 6 weeks. The combined group showed greater improvements in attention and hyperactivity/impulsivity (SNAP-IV) and in executive functions. However, Atomoxetine alone outperformed rTMS in coding and Iowa Gambling Tasks. Overall, combined treatment demonstrated superior therapeutic effects, while medication showed advantages in specific cognitive domains (Cao et al., 2018).

#### 8.11.3 Transcranial direct current stimulation

The clinical investigation of tDCS in ADHD expanded in 2014 with the first double-blind crossover RCT by Prehn-Kristensen et al., which examined the effects of single-session slow-wave oscillatory anodal tDCS over the right and left dorsolateral PFC (dlPFC) in children with ADHD. They found that overnight stimulation, compared to placebo, improved declarative memory in 12 children (Prehn-Kristensen et al., 2014).

In 2015, Munz et al., in a subsequent crossover RCT involving 14 children with ADHD, demonstrated for the first time that single-session slow oscillation-tDCS over the bilateral dlPFC during early non-REM sleep improved behavioral inhibition in Go/No-Go task the following morning, while it had no effects on alertness, digit span, or motor memory (Munz et al., 2015).

In 2015, Soltaninejad et al. conducted a single-blind RCT applying a single session of tDCS over the left dlPFC in 20 high school students with ADHD. Cathodal stimulation improved inhibition accuracy in the Go/No-Go task, while anodal stimulation increased correct responses in the Go condition but had no effect on Stroop interference. These findings provided early evidence that tDCS may enhance inhibitory control in adolescents with ADHD (Soltaninejad et al., 2015).

Also in 2015, Cosmo et al. extended tDCS research to adults with ADHD in the first double-blind RCT examining cognitive control effects of single-session tDCS in adults with ADHD. Participants performed the Go/No-Go task before and after 20 min of anodal tDCS over the left dlPFC; however, the study found no evidence supporting the effectiveness of this treatment for improving inhibitory control in adults with ADHD (Cosmo et al., 2015).

In 2017, Cachoeira et al. conducted the first double-blind RCT examining the potential benefits of tDCS in alleviating symptoms of adults with ADHD using clinical rating scores rather than neuropsychological tasks. Nine adults with ADHD received active tDCS with an anodal right/cathodal left dlPFC montage for 5 days. Compared to the 8 participants in the sham group, active tDCS improved inattention but not hyperactivity/impulsive symptoms immediately post-treatment and at a 2-week follow-up. Total ADHD scores also improved at the 2-week follow-up, although group differences disappeared by the 4-week follow-up (Cachoeira et al., 2017).

#### 8.11.4 External trigeminal nerve stimulation

Research on external trigeminal nerve stimulation (eTNS) began in 2015 with McGough et al.'s open-label feasibility study in children and adolescents with ADHD. eTNS was applied nightly during sleep for 8 weeks. Significant improvements were observed in ADHD symptoms (ADHD-IV), executive functioning (BRIEF), and attention performance, along with reductions in sleep-related problems. The treatment was well-tolerated, with only mild, transient side effects such as eye twitching and headache (McGough et al., 2015).

In 2019, McGough et al. conducted the first double-blind, sham-controlled pilot study of eTNS in 62 children with ADHD. Nightly treatment over 4 weeks significantly improved ADHD symptoms (ADHD-RS) and Clinical Global Impression ratings. Additionally, quantitative EEG revealed increased spectral power in right frontal and midline regions following active eTNS (McGough et al., 2019).

These clinical developments culminated in 2019, when the FDA granted marketing authorization for the Monarch eTNS System through the de novo classification pathway. This was the first medical device authorized for the treatment of ADHD in children aged 7–12 years who are not currently taking prescription ADHD medication. The authorization followed studies by (McGough et al., 2015) and (McGough et al., 2019), which demonstrated the safety and potential efficacy of eTNS. The device delivers low-level electrical stimulation to the trigeminal nerve, modulating brain activity associated with ADHD symptoms (Administration USFaD, 2019).

#### 8.11.5 Photobiomodulation

The clinical investigation of transcranial photobiomodulation (tPBM) in ADHD emerged in 2025, when Lai et al. conducted the first open-label, uncontrolled trial using 1,064 nm stimulation in adults. In this study, 48 participants received seven consecutive daily sessions of tPBM targeting the left PFC. The intervention was associated with improvements in cognitive domains, including working memory, attention, and inhibitory control, with effects peaking approximately 2–3 weeks post-treatment. However, the absence of a sham control group and the lack of neurophysiological measures limit causal inference and mechanistic interpretation (Lai et al., 2025).

In 2026, Salehpour et al. conducted the first single-blind RCT demonstrating the beneficial effects of a single session of 1,064 nm laser stimulation on cognitive functioning and brain hemodynamic responses in adults with ADHD. An 8-min application of tPBM to the right forehead improved impulse control, attentional processing, and working memory, as well as prefrontal oxygenation (Salehpour et al., 2026).

Collectively, scientific developments since 2000 have shifted ADHD research away from single-deficit and categorical accounts toward developmental, network-level, polygenic, and dimensional frameworks. Large-scale neuroimaging and genetic studies, greater recognition of heterogeneity across the lifespan, international consensus, and expanding pharmacological and neuromodulatory treatments have established a more integrative understanding of ADHD and set the stage for contemporary precision psychiatry approaches.

## 9 Conclusions and future directions

### 9.1 Major conceptual transitions and revisions in ADHD

Across more than two centuries, the conceptualization of ADHD has undergone several major paradigm shifts. Early descriptions emphasized disturbances of attention, impulsivity, behavioral control, and, at times, moral explanations of behavior. During the early 20th century, observations of behavioral syndromes following encephalitis lethargica, together with emerging concepts of brain function, increasingly shifted the field toward medical and neurological explanations of ADHD-like behaviors. By the mid-20th century, the emergence of stimulant therapy and the transition from concepts of brain injury and minimal brain damage toward minimal brain dysfunction further strengthened the biological and clinical understanding of the disorder, reflecting growing recognition that functional abnormalities could occur without demonstrable structural lesions (Clements, 1966). These developments also helped lay the foundation for increasingly symptom-based diagnostic frameworks and systematic experimental research. During the 1960s and 1970s, emphasis shifted toward hyperactivity as the defining behavioral feature, followed by increasing recognition of sustained attention and impulse-control deficits (Douglas, 1972). By the 1980s and 1990s, executive dysfunction, behavioral inhibition, motivational processes, and fronto-striatal mechanisms became increasingly prominent in theoretical models (Barkley, 1997). More recently, advances in neuroimaging and genetics have shifted the field away from single-brain-structure or single-deficit explanations toward distributed brain-network, polygenic, and multilevel neurodevelopmental models (Cortese et al., 2012). These broader conceptual transitions were accompanied by substantial changes in ADHD terminology and diagnostic classification (as summarized in Table 1). The major historical transitions in ADHD are summarized in Figure 1.

**Table 1:** Historical timeline of ADHD terminology and diagnostic development.

Year	References	Terminology
1753	Kloekhof, 1753	Fleeting attention
1775	Weikard, 1775	Attention deficit
1798	Crichton, 1798	Disease of attention
1812	Rush, 1812	A syndrome involving inability to focus attention
1850	West, 1850	The nervous child
1859	Neumann, 1859	Hypermetamorphosis
1888	Bourneville, 1888	Mental instability
1892	Allbutt, 1892	Unstable nervous system
1899	Clouston, 1899	Simple hyperexcitability
1902	Still, 1902	Defect of moral control
1917	Lafora, 1917	Unstable child
1923	Ebaugh, 1923	Post-encephalitic behavior disorder
1932	Kramer and Pollnow, 1932	Hyperkinetic disorder
1934	Kahn and Cohen, 1934	Organic drivenness
1947	Strauss and Lehtinen, 1947	Brain-injured child
1953	Statten, 1953	Minimal brain damage
1952	DSM-I (American Psychiatric Association, 1952)	N/A
1957	Laufer et al., 1957	Hyperkinetic impulse disorder
1960	Chess, 1960	Hyperactive child syndrome
1966	Clements, 1966	Minimal brain dysfunction
1968	DSM-II (American Psychiatric Association, 1968)	Hyperkinetic reaction of childhood or adolescence
1980	DSM-III (American Psychiatric Association, 1980)	ADD with or without hyperactivity
1987	DSM-III-R (American Psychiatric Association, 1987)	ADHD, undifferentiated ADD
1994	DSM-IV (American Psychiatric Association, 1994)	ADHD (3 subtypes: predominantly inattentive, predominantly hyperactive/impulsive, combined)
2000	Dsm-Iv-tr, 2000	ADHD (3 subtypes: predominantly inattentive, predominantly hyperactive/impulsive, combined)
2013	DSM-5 (APA, 2013)	ADHD (3 presentations: predominantly inattentive, predominantly hyperactive/impulsive, combined)
2022	DSM-5-TR (American Psychiatric Association, 2022)	ADHD (3 presentations: predominantly inattentive, predominantly hyperactive/impulsive, combined)

**Figure 1:**
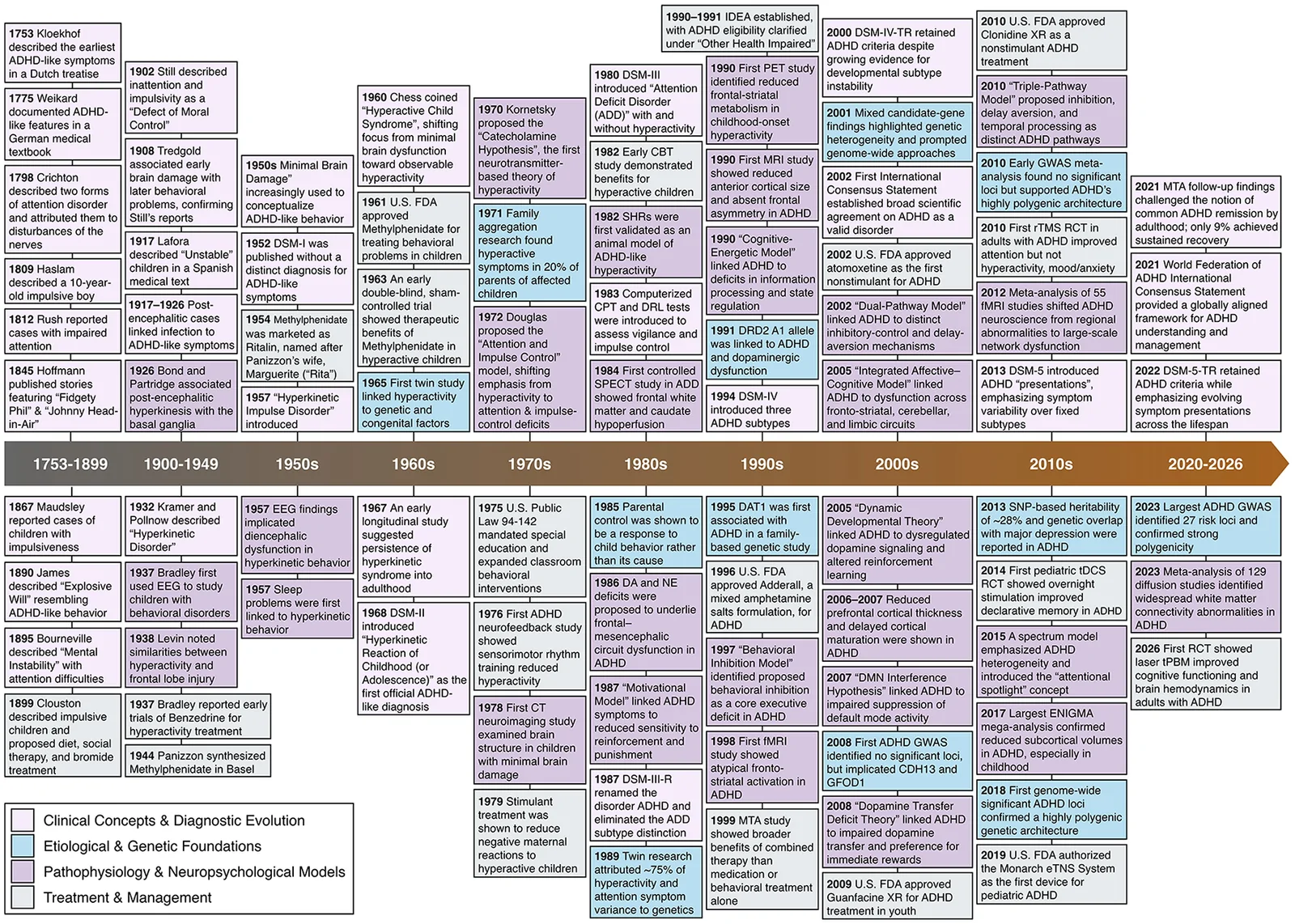
Major milestones and conceptual transitions in ADHD across diagnosis, etiology, genetics, pathophysiology, neuropsychology, treatment, and management from 1753 through 2026.

These conceptual transitions also illustrate how scientific explanations were revised as accumulating evidence challenged earlier assumptions. Although minimal brain dysfunction represented an advance over structural brain injury models, the concept itself gradually declined because of its broad and poorly defined nature, limited diagnostic specificity, and lack of consistent neurological evidence (Lange et al., 2010). Similarly, environmental hypotheses attributing hyperactivity primarily to food additives did not provide a sufficient explanation for the disorder, although subsequent evidence suggests that food additives may influence symptoms in a subset of children with ADHD (Nigg et al., 2012). Early psychodynamic and parenting-based explanations also lost prominence as evidence increasingly indicated that problematic parent–child interactions may partly arise in response to the child's ADHD-related behavior rather than necessarily reflect a primary cause of the disorder (Barkley and Cunningham, 1979).

### 9.2 Contemporary advances shaping precision psychiatry in ADHD

Over the past 50 years, progressive advances in ADHD diagnosis, genetics, neuropsychology, neuroimaging, and treatment have revealed substantial heterogeneity, highlighting the need to integrate multimodal information for individualized prognosis, diagnosis, and treatment (Cortese and Coghill, 2018).

During the 1970s and 1980s, ADHD research increasingly focused on defining its neurobiological, cognitive, and genetic underpinnings. Catecholaminergic theories, particularly involving dopamine and norepinephrine, provided mechanistic explanations for both symptoms and treatment response, while family and twin studies suggested a substantial genetic contribution to the disorder. At the same time, conceptual models evolved from emphasizing hyperactivity alone to recognizing deficits in sustained attention, inhibitory control, and executive functioning as central features. This period also witnessed important advances in behavioral interventions, recognition of ADHD across the lifespan, and growing appreciation of developmental and environmental influences. Successive revisions of the DSM further transformed ADHD into an operationalized diagnosis that could be investigated systematically across clinical and research settings. Together, these developments provided increasingly measurable clinical, cognitive, and biological dimensions through which individual differences in ADHD could be investigated.

The 1990s and early 2000s marked the emergence of a neuroscience-driven era. Structural and functional neuroimaging studies implicated fronto-striatal, fronto-parietal, and cerebellar circuits, reinforcing the view of ADHD as a disorder of atypical neurodevelopment rather than localized dysfunction. Parallel advances in molecular genetics confirmed heritability while revealing the complex contributions of dopaminergic and other neurotransmitter systems. Influential theoretical models expanded beyond single-deficit explanations, incorporating executive dysfunction, motivational processing, temporal regulation, reward sensitivity, and large-scale brain network interactions. These advances also established ADHD as a lifespan neurodevelopmental disorder, characterized by developmental heterogeneity, symptom persistence into adulthood for many individuals, and responsiveness to both pharmacological and multimodal interventions. This convergence of clinical, cognitive, genetic, and neuroimaging evidence increasingly challenged the expectation that a single mechanism could explain ADHD across all individuals and instead highlighted the need to account for biological and clinical heterogeneity.

Over the past two decades, ADHD research has evolved toward increasingly integrative and multidimensional models. Large-scale genome-wide association studies have indicated that ADHD is highly polygenic and shares substantial genetic liability with numerous psychiatric and neurodevelopmental conditions. Concurrent advances in behavioral neuroscience have shifted attention from isolated brain regions to distributed functional networks involved in cognitive control, salience detection, reward processing, and default-mode regulation. Diagnostic concepts have likewise evolved to accommodate developmental changes in symptom expression and dimensional variability across the lifespan, with increasing recognition that ADHD traits may be distributed along a continuum rather than represented solely by discrete diagnostic categories. Adult ADHD has become an increasingly important focus, prompting continued investigation into developmental persistence, late-life manifestations, and the validity of adult-onset presentations. Therapeutic approaches have similarly expanded beyond stimulant medications to include non-stimulant pharmacotherapies, cognitive and behavioral interventions, neurofeedback, and emerging non-invasive neuromodulation approaches, including rTMS, tDCS, eTNS, and prefrontal PBM.

In sum, these contemporary developments show how the historical evolution of ADHD research has progressively established the scientific foundations underlying the current move toward precision psychiatry, with the broader historical progression summarized chronologically in Tables 2–9.

**Table 2:** Historical timeline of influential scientific, clinical, and conceptual developments in ADHD from 1753 to 1949.

Year	Developments
1753	Cornelius Albertus Kloekhof described ADHD-like syndrome in a Dutch psychiatry treatise
1753	Le Camus independently described attention-deficit symptoms in a French medical work, in a manner similar to Kloekhof
1775	Melchior Adam Weikard described ADHD-like syndrome in a German medical textbook
1798	Alexander Crichton described two types of attention disorders in a Scottish medical textbook and attributed them to abnormalities in the nerves
1809	John Haslam described a 10-year-old impulsive boy
1812	Benjamin Rush described three individuals with impaired sustained attention
1845	Heinrich Hoffman published stories of “Fidgety Phil” and “Johnny Head-in-Air”
1867	Henry Maudsley reported cases of children with impulsiveness
1876	David Ferrier was among the first to show that removing the frontal lobe in monkeys led to restlessness, poor concentration, aimless movement, and increased appetite
1890	William James described a personality type with “explosive will” in his book, *The Principles of Psychology*, resembling ADHD-like symptoms
1895	Désiré-Magloire Bourneville described “Mental Instability” in institutionalized children with attentional and behavioral difficulties in French medical texts
1899	Thomas Clouston described impulsive children with learning problems, recommending diet, social therapy, and potassium bromide treatment
1902	George Frederic Still described inattentive and impulsive children in British lectures, referring to it as a “Defect of Moral Control” with a possible biochemical basis
1908	Alfred Frank Tredgold linked early brain damage to later behavioral problems
1917	Gonzalo Lafora described “Unstable” children in a Spanish medical text with ADHD-like syndrome
1917–1926	Encephalitis pandemics established an association between behavioral disorders characteristic of ADHD and infectious disease
1923	Franklin Ebaugh linked post-encephalitic behavioral changes to ADHD-like symptoms
1926	Earl Bond and George Everett Partridge linked post-encephalitic hyperkinetic syndrome to disturbances in the basal ganglia
1932	Franz Kramer and Hans Pollnow described “Hyperkinetic Disorder”
1934	Eugen Kahn and Louis Cohen described “Organic Drivenness” with impulsivity, hyperkinesis, and attention issues; linked to brainstem damage but not limited to encephalitis
1937	Charles Bradley reported the first trials of the stimulant Benzedrine to manage hyperactivity; also first to use EEG in behaviorally disordered children
1938	Paul Levin noted a similarity between children's hyperactivity and behaviors seen in primates with frontal lobe damage
1944	Leandro Panizzon synthesized methylphenidate in Basel
1947	Alfred Strauss and Laura Lehtinen introduced the term “Brain-Injured Child” to describe children exhibiting behavioral problems in the absence of confirmed brain damage

**Table 3:** Historical timeline of influential scientific, clinical, and conceptual developments in ADHD from 1950 to 1969.

Year	Developments
1952	The DSM-I was published and did not include a distinct diagnosis for ADHD-like symptoms or a formal coding system for such presentations
1954	Methylphenidate was marketed as Ritalin, named after Panizzon's wife, Marguerite (“Rita”)
1957	Charles Bradley and Joel Elkes proposed that the effect of amphetamine might be exerted on receptors related to, or possibly located within, the brainstem reticular activating system
1957	Laufer et al. conducted a photo-Metrazol EEG study and proposed a link between diencephalic dysfunction and the hyperkinetic behavior syndrome
1957	Laufer et al. introduced the term “Hyperkinetic Impulse Disorder”
1957	Maurice Laufer and Eric Denhoff suggested, for the first time, a link between sleep disturbances and hyperkinetic behavior
1960	Stella Chess coined “Hyperactive Child Syndrome,” shifting focus from minimal brain dysfunction to observable excessive activity and persistent motion as core features of ADHD
1961	Methylphenidate became the first stimulant to be approved by the FDA for use in children with behavioral problems
1963	Keith Conners and Leon Eisenberg published one of the first double-blind, sham-controlled studies on the therapeutic benefits of Methylphenidate in hyperactive children
1965	Rafael Lopez published the first twin study linking hyperactivity to genetic and congenital factors
1966	A national task force defined minimal brain dysfunction, identifying inattention, impulsivity, and hyperactivity as core symptoms in children with normal intelligence
1967	Menkes et al. reported one of the first longitudinal studies suggesting that hyperkinetic syndrome may persist into adulthood
1967	NIMH funded the first federal research grant to investigate the efficacy of stimulants in children with behavioral problems
1968	A turning point in pediatric psychopharmacology, with grants awarded to Greenberg (UC Davis), Steinberg (DC Mental Health), and Klein (Hillside Hospital)
1968	The DSM-II was published and introduced “Hyperkinetic Reaction of Childhood (or Adolescence)” as the first official diagnosis for ADHD-like symptoms

**Table 4:** Historical timeline of influential scientific, clinical, and conceptual developments in ADHD from 1970 to 1979.

Year	Developments
1970	Conan Kornetsky proposed the first neurotransmitter theory of hyperactivity, the “Catecholamine Hypothesis,” linking it to dysfunctions in catecholaminergic neurotransmission
1971	James Morrison and Mark Stewart conducted one of the first family aggregation studies in hyperactive children, reporting a 20% prevalence of similar symptoms in parents of affected children
1971	Paul Wender proposed the “Minimal Brain Dysfunction” model to explain the core symptoms observed in affected children by 3 main factors: reduced sensitivity to pleasure and pain, an elevated but dysregulated level of arousal, and extraversion
1972	Virginia Douglas proposed an “Attention and Impulse Control” model, positing that impaired sustained attention and impulse control were more probable causes for the challenges observed in ADHD children than mere hyperactivity
1973	Anneliese Pontius proposed that adult hyperactivity and impulsivity in minimal brain dysfunction were linked to frontal lobe and caudate dysfunction, affecting planning and activity regulation
1973	Benjamin Feingold proposed that dietary factors caused over half of hyperactivity cases, suggesting that eliminating certain additives could serve as an effective treatment
1975	The National Advisory Committee on Hyperkinesis and Food Additives (NACHFA) was established to evaluate Feingold's claims, concluding they were largely anecdotal and recommending controlled trials for validation
1975	U.S. Public Law 94–142 was enacted, mandating special education for children with disabilities and expanding support for behavioral interventions in classroom settings
1975	Roberts et al. used a rat lesion model to show that amphetamine-induced hyperactivity was dependent on dopamine but not norepinephrine, offering early experimental evidence linking dopamine dysregulation to hyperactivity
1976	Joel Lubar and Margaret Shouse reported the first neurofeedback study in ADHD, showing that sensorimotor rhythm training reduced hyperactivity, laying the foundation for EEG-based interventions
1978	Humphries et al. emphasized that negative parenting behaviors were more likely reactions to children's poor self-control than causes of hyperactivity
1978	Kjell Bergström and Bo Bille published the first neuroimaging study using CT to examine brain structure in children with minimal brain damage
1979	Russell Barkley and Cunningham showed that stimulant treatment reduced mothers' negative reactions to hyperactive children

**Table 5:** Historical timeline of influential scientific, clinical, and conceptual developments in ADHD from 1980 to 1989.

Year	Developments
1979–1983	Virginia Douglas refined her model of hyperactivity, identifying four key deficits in ADHD: attention regulation, impulse control, arousal modulation, and preference for immediate rewards
1980	The DSM-III was published and introduced “Attention Deficit Disorder (ADD)” with two subtypes: with hyperactivity (ADD+H) and without hyperactivity (ADD–H)
1981	Schachar et al. differentiated between “pervasive” hyperactivity, observed at both home and school, and “situational” hyperactivity, present in only one setting
1982	Philip Kendall and Lauren Braswell published one of the first foundational studies demonstrating the effectiveness of CBT for hyperactive children
1982	Myers et al. for the first time validated spontaneously SHRs as a hyperactivity model for ADHD, showing elevated activity compared to normotensive control rats
1983	Michael Gordon introduced a compact computerized tool that administered two ADHD-sensitive tests, a CPT for vigilance and impulsivity and a DRL test for impulse control
1983	Shaywitz et al. conducted one of the first controlled CT studies in children with ADD, but found no significant structural differences
1984	Lou et al. conducted the first controlled SPECT study in ADD, showing hypoperfusion in the frontal white matter and caudate nuclei, with methylphenidate increasing perfusion in the mesencephalon and basal ganglia
1985	Barkley et al. showed that parental control in ADHD was a response to child behavior, not its cause, and that methylphenidate affected both symptoms and parent-child interactions
1986	Nasrallah et al. conducted a second controlled CT study in hyperkinetic/minimal brain dysfunction, reporting cortical and cerebellar atrophy
1986	Alan Zametkin and Judith Rapoport proposed dopamine and norepinephrine deficits in ADHD, linking frontal–mesencephalic circuit dysfunction to core symptoms
1987	Marianne Haenlein and William Caul proposed the “Motivational Model” of ADHD, suggesting that core symptoms arise from reduced sensitivity to reinforcement and punishment, highlighting altered reward processing mechanisms rather than primary attentional deficits
1987	The DSM-III-R renamed the disorder to ADHD and eliminated the distinction between ADD subtypes, removing ADD–H as a separate category
1988	An international symposium led by Sergeant set ADHD research criteria: multi-setting symptoms, ≥3 of 4 activity/attention issues, onset before age 7, duration ≥2 years, high parent–teacher ratings, and exclusion of autism and psychosis
1989	Robert Goodman and Jim Stevenson conducted one of the first twin studies, estimating that genetic factors account for ~75% of the variance in hyperactivity and attention symptoms
1989	Lou et al. conducted the first controlled SPECT study identifying striatal hypoperfusion as a core pathophysiological feature of ADHD

**Table 6:** Historical timeline of influential scientific, clinical, and conceptual developments in ADHD from 1990 to 1999.

Year	Developments
1990-1991	U.S. Public Law 94–142 was reauthorized as the IDEA, allowing students with ADHD to qualify for special education under the “Other Health Impaired” category
1990	Zametkin et al. published the first PET study using [^1^8F]FDG to demonstrate decreased metabolic activity in the frontal and striatal regions of adults with childhood-onset hyperactivity
1990	Hynd et al. conducted the first MRI study, revealing reduced anterior cortical size, predominantly in the right hemisphere, and absent right-left frontal asymmetry in children with ADHD
1990	Joseph Sergeant proposed the “Cognitive-Energetic Model” or “State Regulation Deficit Model” for ADHD, based primarily on deficiencies in information processing
1990–1995	Biederman et al. reported that up to 35% of immediate family members of children with ADHD were also diagnosed, and if a parent had ADHD, the offspring's risk was ~57%
1991	Comings et al. linked the DRD2 A_1_ allele to ADHD, suggesting dopaminergic dysfunction, especially in cases with comorbid externalizing disorders
1992	Gilger et al. found ADHD concordance was 81% in identical twins and 29% in fraternal twins
1994	The DSM-IV changed the diagnosis to ADHD with three subtypes (predominantly hyperactive, predominantly inattentive, and combined) and, for the first time, acknowledged that most individuals experience symptoms into late adolescence and adulthood
1995	Cook et al. reported the first genetic association between the DAT1 and ADHD in a family-based study
1996	LaHoste et al. found the DRD4 seven-repeat allele overrepresented in children with ADHD
1996	Adderall, a mixed amphetamine salts formulation (amphetamine and dextroamphetamine), was approved by the FDA for the treatment of ADHD
1997	Russell Barkley proposed the “Behavioral Inhibition Model” of ADHD, identifying behavioral inhibition as the core executive deficit in ADHD, primarily applicable to hyperactive-impulsive and combined presentations
1997	Russell Barkley criticized the view proposed by Hill and Schoener (1996) that ADHD rates decline sharply into adulthood, calling it a statistical illusion
1998	Vaidya et al. conducted the first fMRI study in ADHD, showing atypical fronto-striatal activation; methylphenidate increased frontal and striatal activation and improved response inhibition
1999	The original MTA study, funded by NIMH, showed that combined therapy offered broader benefits than medication or behavioral treatment alone

**Table 7:** Historical timeline of influential scientific, clinical, and conceptual developments in ADHD from 2000 to 2009.

Year	Developments
2000	DSM-IV-TR was introduced and retained ADHD criteria, but emerging evidence challenged subtype validity, highlighting developmental instability and supporting a dimensional model of symptom severity
2000	Joseph Sergeant updated the “Cognitive-Energetic Model” to incorporate cognitive strategies; impulsivity was redefined as fast, inaccurate information processing due to suboptimal resource allocation
2001	Early candidate gene studies of CHRNA4, ADRA2A, and 5-HTTLPR in ADHD yielded mixed findings, highlighting genetic heterogeneity and motivating the shift to genome-wide approaches
2002	Barkley et al. published the first International Consensus Statement on ADHD, establishing broad scientific agreement on its validity as a disorder
2002	Atomoxetine was approved by the FDA as the first non-stimulant for ADHD, offering a non-scheduled alternative to stimulants and becoming a widely recommended second-line treatment
2002	Castellanos et al. reported one of the earliest longitudinal MRI studies showing reduced caudate nucleus volume in children with ADHD, supporting fronto-striatal dysfunction and delayed subcortical development
2002	Edmund Sonuga-Barke proposed the “Dual-Pathway Model” of ADHD, distinguishing impaired inhibitory control and delay aversion as independent mechanisms linked to mesocortical and mesolimbic dopamine systems
2003	El-Sayed et al. proposed the “Maturational Lag Hypothesis,” suggesting ADHD reflects delayed CNS development, with children showing younger-like neurodevelopmental patterns that may partially normalize with age
2004	The MTA 24-month follow-up showed a sustained but reduced advantage of medication over behavioral and community care, with no added benefit of combination therapy beyond medication alone
2005	Edmund Sonuga-Barke argued for multiple developmental pathway models of ADHD, integrating cognitive and motivational processes and emphasizing environmental influences, challenging single-deficit theories
2005	Virginia Douglas proposed the “Self-Regulation Model,” identifying deficits in effortful attention, inhibitory control, and strategic processes, emphasizing ADHD heterogeneity and the need for component-specific assessment
2005	Joel Nigg and BJ. Casey proposed an “Integrated Affective–Cognitive Model,” linking ADHD to dysfunction across fronto-striatal, cerebellar, and limbic circuits, integrating cognitive control and emotional processing
2005	Sagvolden et al. proposed the “Dynamic Developmental Theory,” linking ADHD to dysregulated tonic and phasic dopamine signaling, with mesolimbic hypofunction altering reinforcement learning and behavioral regulation
2006	Jeffrey Halperin and Kurt Schulz proposed a “Neurodevelopmental Model,” suggesting ADHD arises from early non-cortical dysfunctions, with executive deficits emerging secondarily and improving with development
2006–2007	Shaw et al. demonstrated regionally reduced cortical thickness in prefrontal regions and, critically, a delay in cortical maturation in children with ADHD, establishing a developmental neuroanatomical model of the disorder
2007	Edmund Sonuga-Barke and Francisco Castellanos proposed the “DMN Interference Hypothesis,” suggesting ADHD reflects impaired suppression of default mode activity, disrupting task networks and causing attentional lapses
2008	Neale et al. conducted the first GWAS of ADHD, identifying no genome-wide significant loci for diagnosis, but linking CDH13 and GFOD1 to ADHD-related quantitative traits
2008	Gail Tripp and Jeff Wickens proposed the “Dopamine Transfer Deficit Theory,” suggesting impaired transfer of dopamine signaling to predictive cues leads to reduced anticipatory reinforcement and a bias toward immediate rewards in ADHD
2009	Guanfacine XR was approved by the FDA as a non-stimulant for ADHD in youth, targeting impulsivity and emotional regulation as an alpha2 agonist

**Table 8:** Historical timeline of influential scientific, clinical, and conceptual developments in ADHD from 2010 to 2019.

Year	Developments
2010	The European Consensus Statement on adult ADHD was published
2010	Clonidine extended-release was approved by the FDA as a non-stimulant for ADHD, providing an alpha-2 agonist option for managing hyperactivity, impulsivity, and attention symptoms
2010	Edmund Sonuga-Barke introduced the “Triple-Pathway Model,” adding temporal processing deficits as a third independent pathway alongside inhibition and delay aversion, highlighting dissociable neuropsychological mechanisms in ADHD
2010	Neale et al. conducted one of the first GWAS meta-analyses in ADHD, identifying no genome-wide significant loci but supporting a highly polygenic architecture and emphasizing the need for larger collaborative studies
2010	Bloch et al. conducted the first single-blind crossover RCT of rTMS in adults with ADHD, showing improved attention but no effects on hyperactivity, mood, or anxiety
2011	Durston et al. supported the multi-pathway framework, proposing separable circuits for cognitive control, reward, and timing, and introducing vigilance deficits as a fourth neurocognitive component in ADHD
2012	Weaver et al. conducted the first multi-session RCT of rTMS in ADHD, showing safety and overall improvement but no differences between active and sham conditions
2012	Cortese et al. conducted a landmark meta-analysis of 55 fMRI studies, showing that ADHD involves dysfunction across large-scale networks, shifting the field from regional abnormalities toward a systems-level neuroscience framework
2013	DSM-5 introduced ADHD “presentations,” emphasizing symptom fluidity and variability
2013	Russell Barkley proposed the term Concentration Deficit Disorder to replace Sluggish Cognitive Tempo, aiming to improve conceptual clarity and reduce negative connotations
2013	The Psychiatric Genomics Consortium reported SNP-based heritability of ~28% for ADHD and identified genetic overlap with major depression, advancing understanding of shared psychiatric risk
2013	Arnold et al. conducted a double-blind RCT of neurofeedback in ADHD, showing similar improvements in active and sham groups, with no added benefit of higher session frequency
2014	Prehn-Kristensen et al. conducted the first double-blind crossover RCT using tDCS in children with ADHD, showing that overnight stimulation improved declarative memory
2015	Cosmo et al. conducted the first double-blind RCT of tDCS in adults with ADHD, finding no improvement in inhibitory control following single-session stimulation
2015	Rebeca Heidbreder proposed a spectrum model of ADHD, emphasizing heterogeneity and introducing the “attentional spotlight” concept to explain variability across symptom presentations
2017	The ENIGMA ADHD Working Group conducted the first largest mega-analysis of subcortical volumes, confirming reductions in key regions, particularly in childhood, and supporting the neurodevelopmental model of ADHD
2017	Kotov et al. introduced HiTOP, embedding ADHD within the externalizing spectrum and framing it as a dimensional construct with shared mechanisms and high comorbidity across psychopathology
2018	The ADHD Working Group of the Psychiatric Genomics Consortium identified the first genome-wide significant loci for ADHD, confirming its polygenic architecture and advancing biological and therapeutic insights
2018	Cao et al. conducted a single-blind RCT comparing rTMS and Atomoxetine in children with ADHD, showing superior outcomes with combined treatment and domain-specific advantages for medication
2019	McGough et al. conducted the first double-blind, sham-controlled eTNS study in ADHD, showing symptom improvement and increased frontal EEG activity with active treatment
2019	The FDA authorized the Monarch eTNS System, the first device for pediatric ADHD

**Table 9:** Historical timeline of influential scientific, clinical, and conceptual developments in ADHD, 2020–2026.

Year	Developments
2021	The MTA follow-up showed only 9% of individuals achieved sustained recovery, while most exhibited fluctuating remission and recurrence, challenging the notion that ADHD commonly remits by adulthood
2021	The World Federation of ADHD International Consensus Statement was published, providing the most comprehensive and globally aligned framework for ADHD understanding and management
2022	DSM-5-TR was introduced, retaining ADHD criteria while emphasizing its dynamic nature and evolving symptom presentations across the lifespan
2022	An international expert group proposed the term Cognitive Disengagement Syndrome to replace Sluggish Cognitive Tempo, aiming for greater accuracy and reduced stigma
2023	Parlatini et al. conducted a meta-analysis of 129 diffusion imaging studies, showing reduced fractional anisotropy across major white matter pathways, highlighting large-scale connectivity abnormalities in ADHD
2023	Demontis et al. conducted the largest GWAS of ADHD, identifying 27 risk loci, confirming strong polygenicity and genetic overlap with other psychiatric disorders, and linking risk variants to frontal and dopaminergic brain systems
2025	Lai et al. reported the first open-label, uncontrolled trial showing that 1,064 nm tPBM targeting the left PFC improved cognitive function in adults with ADHD, with peak effects at 2–3 weeks post-treatment
2026	Salehpour et al. conducted the first single-blind RCT demonstrating the beneficial effects of 1,064 nm laser tPBM on cognitive functioning and brain hemodynamic responses in adults with ADHD

### 9.3 Future directions and remaining challenges

Despite remarkable progress, several important challenges remain that are likely to shape the next generation of ADHD research. Perhaps the most significant is the continued transition from categorical diagnostic models toward dimensional and biologically informed conceptualizations that better capture the substantial heterogeneity observed across individuals. Integrating behavioral phenotypes with cognitive, neurophysiological, neuroimaging, and genetic measures will be essential for developing mechanistically informed classifications capable of supporting precision diagnosis and individualized treatment.

Equally important is the need to better understand ADHD across the lifespan. Although adult ADHD is now widely recognized, fundamental questions remain regarding developmental trajectories, mechanisms underlying symptom persistence or remission, age-related changes in neurobiology, and the validity of proposed adult-onset presentations. Longitudinal studies spanning childhood through older adulthood will be critical for resolving these questions.

Another major priority is the identification of clinically meaningful biomarkers. Advances in neuroimaging, digital phenotyping, cognitive assessment, and multi-omics technologies offer unprecedented opportunities to characterize ADHD at multiple levels of analysis. However, no single biomarker has yet demonstrated sufficient diagnostic accuracy or reproducibility for routine clinical application. Future progress will likely depend on multimodal approaches that integrate complementary biological and behavioral measures using advanced computational and machine-learning methods.

Future research must also improve the representativeness of ADHD populations. Historically, the predominance of male pediatric samples has limited understanding of sex-specific developmental trajectories, symptom expression, and treatment response. Greater inclusion of females, adults, older individuals, and diverse racial, ethnic, and socioeconomic populations will substantially improve the generalizability of future discoveries.

Finally, increasing attention should be directed toward attentional conditions that extend beyond traditional ADHD boundaries, including CDS and related non-hyperactive attentional phenotypes. Determining whether these represent distinct disorders, overlapping dimensions, or alternative developmental pathways may fundamentally reshape future nosology and mechanistic models of attention disorders. At the same time, continued investigation of non-pharmacological interventions such as non-invasive neuromodulation, behavioral therapies, cognitive training, and combination approaches offers promising opportunities to develop more personalized, mechanism-based treatments.

As ADHD research enters the era of precision neuroscience, its greatest challenge is no longer demonstrating that ADHD is biologically based but rather understanding how diverse biological mechanisms interact across development to produce the heterogeneous clinical presentations observed in practice. Addressing this challenge will require increasingly interdisciplinary research integrating systems neuroscience, developmental psychology, genetics, computational modeling, and clinical science. Such an approach holds the greatest promise for transforming ADHD from a symptom-based diagnosis into a biologically informed, personalized neurodevelopmental disorder with more precise diagnostic tools and targeted therapeutic strategies.
